# Development of a Multi-Epitope Universal mRNA Vaccine Candidate for Monkeypox, Smallpox, and Vaccinia Viruses: Design and In Silico Analyses

**DOI:** 10.3390/v15051120

**Published:** 2023-05-07

**Authors:** Nino Rcheulishvili, Jiawei Mao, Dimitri Papukashvili, Shunping Feng, Cong Liu, Xidan Yang, Jihui Lin, Yunjiao He, Peng George Wang

**Affiliations:** 1 Department of Pharmacology, School of Medicine, Southern University of Science and Technology, Shenzhen 518000, China; nino@sustech.edu.cn (N.R.); maojw@sustech.edu.cn (J.M.); dimitri@sustech.edu.cn (D.P.); 12133106@mail.sustech.edu.cn (S.F.); 11930759@mail.sustech.edu.cn (C.L.); xidanyang993@163.com (X.Y.); linjihui@swmu.edu.cn (J.L.); 2 School of Nursing, Southwest Medical University, Luzhou 646000, China

**Keywords:** monkeypox, mpox, MPXV, universal vaccine, multi-epitope mRNA vaccine, immunoinformatics

## Abstract

Notwithstanding the presence of a smallpox vaccine that is effective against monkeypox (mpox), developing a universal vaccine candidate against monkeypox virus (MPXV) is highly required as the mpox multi-country outbreak has increased global concern. MPXV, along with variola virus (VARV) and vaccinia virus (VACV), belongs to the Orthopoxvirus genus. Due to the genetic similarity of antigens in this study, we have designed a potentially universal mRNA vaccine based on conserved epitopes that are specific to these three viruses. In order to design a potentially universal mRNA vaccine, antigens A29, A30, A35, B6, and M1 were selected. The conserved sequences among the three viral species—MPXV, VACV, and VARV—were detected, and B and T cell epitopes containing the conserved elements were used for the design of the multi-epitope mRNA construct. Immunoinformatics analyses demonstrated the stability of the vaccine construct and optimal binding to MHC molecules. Humoral and cellular immune responses were induced by immune simulation analyses. Eventually, based on in silico analysis, the universal mRNA multi-epitope vaccine candidate designed in this study may have a potential protection against MPXV, VARV, and VACV that will contribute to the advancement of prevention strategies for unpredictable pandemics.

## 1. Introduction

Unforeseeable outbreaks of infectious diseases are causing a rise in the worldwide risk to public health. Monkeypox virus (MPXV) has emerged in May 2022 and affected more than 86,000 people until now [1]. Monkeypox (mpox) is a zoonotic disease that has been largely neglected although there were cases in its endemic areas—West and Central Africa. MPXV, along with variola virus (VARV), vaccinia virus (VACV), and cowpox virus, belongs to the genus *Orthopoxvirus*, family Poxviridae. MPXV is a large, 200–300 nm, brick-shaped virus that exists in two different infectious forms: extracellular enveloped virions (EVs) and intracellular mature virions (MVs). EVs have an extra envelope compared to MVs. The genome of MPXV is about 197 kb linear double-stranded DNA (dsDNA) that encodes approximately 190 proteins [2,3,4]. Due to their intricate structure, many viral antigens and their functions still need to be studied.

Except for Africa, there was an outbreak in the United States (US) in 2003 when imported prairie dogs from Ghana spread the virus, and, consequently, there were 47 confirmed cases [5,6]. Interestingly, the prairie dogs were housed with African rodents, so the virus could have been transmitted via the rodents [7]. In addition, single cases of mpox were identified in different countries such as the US [8,9], Israel, the United Kingdom (UK), and Singapore and all were linked to travel to Nigeria [10]. What caused the MPXV virus outbreak this time? This and many other questions are unanswered until now. However, it is assumed that eradicating smallpox and ceasing vaccination in 1980 globally led to increased cases of mpox. This theory is quite convincing as more than 70% of people are unvaccinated against the smallpox virus today, and most cases of mpox take place in younger people. Interestingly, most of the cases occur in men who have sex with men (MSM), and close physical contact plays a key role in transmission [11,12,13]. Indeed, the sex-related infection rate was always observed even before the current outbreak—males predominated over females, while children accounted for the majority of cases [7]. The fatality rates range from 1% to 11% [14], and the disease is more severe in young children [15,16]. There are two genetic clades of MPXV: the Central African (Democratic Republic of the Congo (DRC), previously known as Zaire) and the West African clades. Out of these two clades, the Central African clade is more virulent and deadly. Unlike the coronavirus disease 2019 (COVID-19) and influenza viruses, the MPXV is a dsDNA [17] virus and is more stable. Generally, the mutation rate of DNA viruses is much lower than RNA viruses. Therefore, the assumption of an MPXV mutation as the only reason for the current outbreak would not be rational. However, a few mutations still take place, and it should not be completely neglected. Even though a smallpox vaccine is quite effective against the MPXV virus, there is still a necessity for developing a new, universal vaccine based on conserved elements. Moreover, apparently, the smallpox vaccine cannot completely protect from MPXV [18]. Additionally, in the worst-case scenario, if certain mutations take place in MPXV and the effectiveness of the smallpox vaccine to MPXV decreases, the situation will also worsen. Although the daily confirmed cases of mpox are significantly decreasing, it is globally spread, and the re-emergence of the same clade or even the spread of the Central African clade is anticipated. Remarkably, messenger RNA (mRNA)-based vaccines have revolutionized the field of vaccinology due to their favorable safety profile, low-cost manufacturing, high potency, and rapid development among other impressive advantages [19,20,21]. mRNA vaccine contains the antigen sequence that is translated into the corresponding protein after the introduction into the host body. After the antigen is released, it is recognized by antigen-presenting cells (APCs) and phagocytosed. The vaccine antigen is then processed into small peptides that are presented on the cell surface via major histocompatibility complex (MHC) I and II, and the cellular and humoral immune responses are induced [20].

In this research, five antigen proteins were selected from MPXV, VACV, and VARV. The corresponding sequences were retrieved and aligned. Conserved sequences were detected in each protein. Relevant T cell epitopes that were tested through experimentation were searched in the immune epitope database (IEDB), sorted according to the optimal results, and selected, while B cell epitopes were predicted via IEDB due to the lack of B cell epitope data in the database. The multi-epitope mRNA vaccine made up of the conserved epitopes of A29, A30, A35, B6, and M1 proteins (encoded by the genes *A29L*, *A30L*, *A35R*, *B6R*, and *M1R*, respectively) was constructed. Various properties of the vaccine were assessed in silico. Based on the obtained results, the mRNA construct proposed in this study has potentially high efficiency and elicits protection from MPXV, VARV, and VACV. The proposed construct represents a favorable candidate for developing the mRNA vaccine for global purposes. The contribution of the design of this potentially universal vaccine candidate is one step forward in the advancement of vaccine development and pandemic alertness.

## 2. Methods

### 2.1. Antigen Selection

Five antigens that are common for MPXV, VARV, and VACV were selected: A29 (A30 in VARV, A27 in VACV), A30 (A31 in VARV, A28 in VACV), A35 (A36 in VARV, A33 in VACV), M1 (M1 in VARV, L1 in VACV), and B6 (B7 in VARV, B5 in VACV). These proteins are highly conserved among the orthopoxviruses and serve important functions in immune response [3,22,23,24,25,26,27]. All five antigens were selected based on their functions in immune response and their successful application in previous vaccine studies [24,27,28,29]. The selected antigens along with their functions are given in Table 1.

### 2.2. Selection of Conserved Regions and Epitopes

The amino acid sequences of A29, A30, A35, B6, and M1 of MPXV and the corresponding proteins of VARV and VACV were retrieved from the National Center for Biotechnology Information (NCBI) database in FASTA format. In total, 372 sequences were downloaded for the antigen A29: 159 A29 (MPXV), 120 A30 (VARV), and 93 A27 (VACV). This protein is 110 amino acids in length, thus, the sequences longer or shorter than 110 amino acids were removed, and the rest (176 sequences) were aligned together. A total of 433 sequences were retrieved for the antigen A30: 363 A30 (MPXV), 8 A31 (VARV), and 62 A28 (VACV). As the full length of this protein is 146 amino acids, all the shorter or longer sequences were removed, and the remaining 158 sequences were aligned together. A total of 365 sequences of the antigen A35 were downloaded: 153 A35 (MPXV), 109 A36 (VARV), and 103 A33 (VACV). As the full-length sequence of A35 is 181 amino acids, A36 is 184, and A33 is 185, all the records shorter than 181 and longer than 185 were removed, and the remaining 194 sequences were aligned. In total 365 sequences of antigen B6 were retrieved: 180 B6 (MPXV), 6 B7 (VARV), and 179 B5 (VACV). The full length of this protein is 317 amino acids, thus, all the sequences shorter or longer than 317 amino acids were removed, and the rest of the sequences (196) were aligned. A total of 391 sequence records of antigen M1 were retrieved from NCBI: 82 M1 (MPXV), 6 M1 (VARV), and 303 L1 (VACV), which were aligned together. As the full length of this protein is 250 amino acids, all the sequences shorter or longer than 250 were removed, and the remaining 346 sequences were aligned together. The conserved sequences were identified via the bioinformatics software Jalview 2.11.1.4 [30]. The filter of conservation threshold was given 10 “below threshold”.

Experimentally tested T cell epitopes and MHC ligands of each antigen were found on IEDB [31] according to the species of orthopoxviruses (MPXV, VARV, and VACV) and host (human). IEDB is an excellent database supported by experimental data of humans and animals on antibody and T cell epitopes. IEDB also incorporates immunoinformatics tools for epitope prediction [31]. As there were no B cell epitopes found on IEDB, the Bepipred linear epitope prediction tool (v2.0) was used to predict B cell epitopes [32]. Ultimately, experimentally tested T cell epitopes were retrieved from IEDB; epitopes containing conserved sequences were ranked and the most studied ones were selected. On the other hand, linear B cell epitopes were predicted using the consensus sequence of each antigen.

### 2.3. Vaccine Design

For the construction of the multi-epitope mRNA vaccine, the epitopes were arranged orderly. B cell epitopes were joined with a flexible linker KK [33,34], while a GGGS linker was used to connect the T cell epitopes [35]. The leading sequence of tissue plasminogen activator (tPA) that augments the antigen presentation [36] was used as a signal sequence (MDAMKRGLCCVLLLCGAVFVSPS). The incorporation of the amino acid sequence of interleukin 6 (IL-6) was done to enhance the immunogenicity of the vaccine [37]. The pan-HLA DR binding epitope (PADRE sequence) was connected to the IL-6 sequence via a GGGS linker and to an epitope of the B cell via EAAAK [34,38]. A polyhistidine (6x) tag was placed on the C-terminal of the vaccine sequence for facilitating the fusion protein detection [39]. The complete open reading frame of the proposed vaccine is given in the Supplementary Materials.

### 2.4. Prediction of Vaccine Properties 

To check whether the antigen could provoke an allergic response, the AlgPred server, a highly accurate tool, was used to predict its allergenicity [40]. As the prediction tool, a hybrid approach—Support Vector Machine (SVMc) algorithm + IgE epitope + ARPs BLAST + MAST—was selected [40,41]. This method detects the allergenicity of the protein based on the composition of amino acids and dipeptides using SVM, which is a motif-based technique using the software MAST. Ultimately, the tool specifies the antigen as an allergen if it contains a segment identical to the known IgE epitopes or similar to allergen-representative proteins [40,41]. For the prediction of antigenicity, server Vaxijen v2.0, which is based on an alignment-independent prediction of protective antigens, was used [42]. Vaxijen categorizes viral, bacterial, and tumor antigens according to their physicochemical characteristics [43]. 

ProtParam was used to calculate the physicochemical properties [44]. The following parameters were characterized: the molecular weight (MW) of the multi-peptide, the atomic and amino acid composition, the theoretical isoelectric point (pI), the instability, the estimated half-life, the grand average of hydropathicity (GRAVY), and the aliphatic indexes. MW was calculated by adding the average isotopic masses of amino acids and the average isotopic mass of one water molecule. In addition to the MW, the pI, which plays a crucial role in pH-dependent properties, was computed based on the pKa value of amino acids, which plays an essential role in characterizing the pH-dependency of the protein. The estimated half-life denotes the duration required from the synthesis of the protein until its decay and reduction to half of its original amount within the cell. The instability index is used to evaluate the stability of the protein in a test tube. An instability index of less than 40 is considered an indication of stability. When this measure is >40, the protein of interest is considered unstable. To compute GRAVY, the hydropathy values of each amino acid are summed up and then divided by the total residue number in the protein. A higher number denotes that the amino acids are more hydrophobic [44]. An aliphatic index is used to characterize the protein relative volume that is taken up by amino acids that have aliphatic side chains, and it is considered a positive factor in augmenting its thermostability.

### 2.5. Tertiary Structure Prediction and Evaluation of Its Quality; Discontinuous B Cell Epitope Prediction 

RoseTTAFold was used to generate the tertiary structure of the vaccine protein [45]. By combining the sequence data of a one-dimensional protein with the two-dimensional data of the distances between the amino acids, as well as the prediction of the three-dimensional atomic structure, this tool is able to forecast the configuration and interplay of the protein [45]. After preparing the structure of the vaccine protein, it was refined with GalaxyRefine [46]. Through the use of molecular dynamics simulation, this immunoinformatics approach reconstructs and reorganizes the amino acid side chains, while also relaxing the overall protein structure [46]. Subsequently, the Ramachandran plot and ERRAT were used to verify the quality of the protein tertiary structure. The Ramachandran plot visualizes the energetically allowed regions for backbone dihedral angles ψ against φ of amino acid residues in protein structures, which allows the testing of the quality of protein structures [47]. The ERRAT score serves as a quality indicator for the non-bonded interactions, where a larger score suggests a superior quality of the protein tertiary structure. ElliPro was employed for the prediction of B cell conformational epitopes based on the 3D structure of the protein [48]. 

### 2.6. Immune Simulation 

Humoral and cellular immune responses driven against the proposed multi-epitope vaccine protein were analyzed via the C-ImmSim server (https://kraken.iac.rm.cnr.it/C-IMMSIM/; accessed on 31 August 2022). This immunoinformatics approach provides the service of immune simulation and characterizes the immune system responses, both humoral and cellular, towards vaccination. For predicting immune epitopes and analyzing immune interactions, C-ImmSim applies a position-specific scoring matrix in combination with machine learning techniques. After uploading the sequence of vaccine antigen in FASTA format, the server predicted the immune responses [49]. The “Allele Frequency Net Database” identified the most widespread HLA-A, HLA-B, and DRB alleles globally. The outcome indicated HLA-A*02:01, HLA-A*01:01, HLA-B*07:02, HLA-B*08:01, DRB1*07:01, and DRB1*15:01. The vaccine was administered thrice with an interval of four weeks between each injection. The simulation volume was adjusted at 10, and the simulation progressed through 270 steps. The vaccine used during the simulation did not comprise LPS. The random seed was 12345, and time periods were set at 1, 85, and 169 [50].

### 2.7. In Silico Validation of Vaccine Protein Binding to the Host Receptors 

In order to assess the binding capacity of vaccine protein to antigen recognition receptors, molecular docking was carried out with a ClusPro server. ClusPro calculates the docking interaction between two protein structures and provides a list of potential complexes in order of priority. It is performed based on a predicted conformation of ligand, orientation, and position as well as binding affinity analysis. Ultimately, the complexes that exhibit good electrostatic and desolvation energies are chosen [51]. A molecular docking of vaccine construct with MHC-I (HLA-A*02:01) (6TDS) and MHC-II (HLA-DRB1*01:01) (1AQD) host receptors was carried out. The following epitopes of the vaccine construct were docked with the MHC molecules: TLFPGDDDL (A29/A30/A27), NTLSERISSK (M1/M1/L1)—MHC-I ligands; FFIVVATAAVCLLFI (A30/A31/A28), LSMITMSAFLIVRLN (A35/A36/A33), ASYISCTANSWNVIP (B6/B7/B5), and KIQNVIIDECY (M1/M1/L1)—MHC-II ligands. The tertiary structure of these epitopes of the vaccine construct was predicted with AlphaFold2 [52,53]. The PDB files 6TDS and 1AQD were edited and cleaned to remove heteroatoms, bound peptides, and water molecules. 

## 3. Results

### 3.1. Selecting Conserved Epitopes

**Figure 1:**
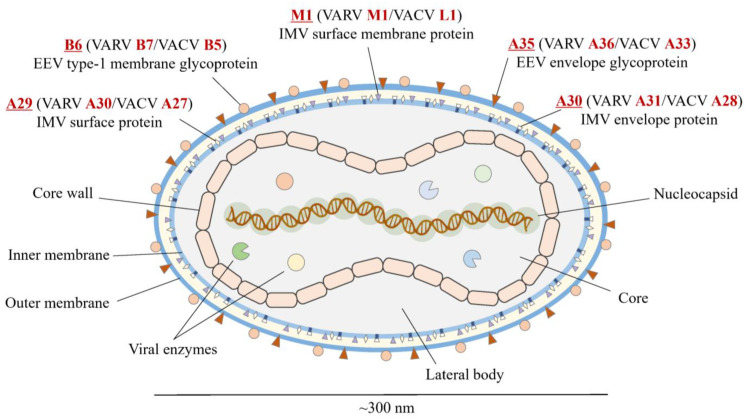
Structure of MPXV and selected viral antigens. Red font indicates the names of the antigens in MPXV, VARV, and VACV. Underline indicates the special emphasis on the name of antigen in MPXV. MPXV, monkeypox virus; VARV, variola virus; VACV, vaccinia virus.

**Figure 2:**
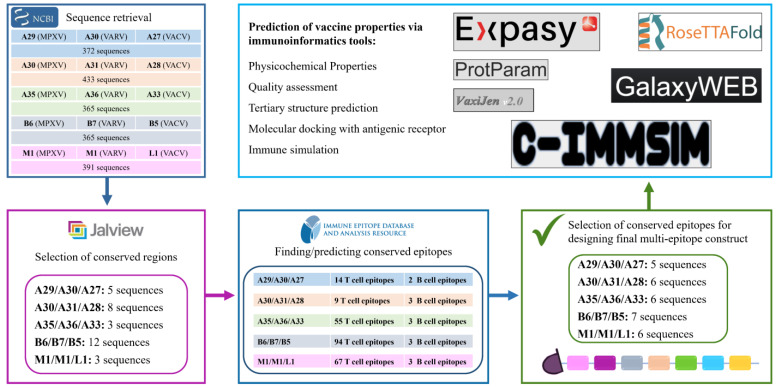
Strategy of designing a potentially universal MPXV mRNA vaccine. MPXV, monkeypox virus. MPXV, monkeypox virus; VARV, variola virus; VACV, vaccinia virus.

**Figure 3:**
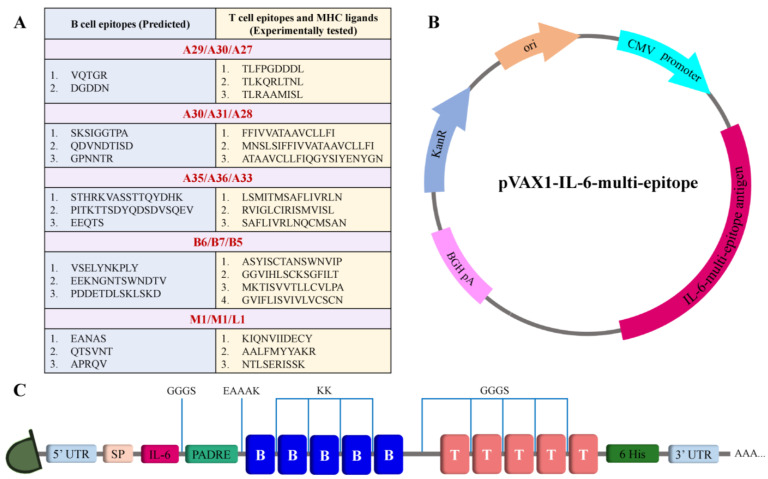
Schematic illustration of the designed mRNA and plasmid. (**A**) Table of selected conserved epitopes for the mRNA vaccine design. (**B**) Plasmid scheme. (**C**) Final design of the multi-epitope mRNA vaccine construct.

The selected antigens in the virus are illustrated in Figure 1. All the conserved sequences and epitopes of each antigen are given in the Supplementary Materials (Tables S1–S10). The strategy for designing a potentially universal MPXV mRNA vaccine based on the conserved epitopes is given in Figure 2. The ultimately selected epitopes that were used for the vaccine design along with the MPXV multi-epitope universal mRNA vaccine construct and the plasmid vector are given in Figure 3.

### 3.2. Assessment of Structure

**Figure 4:**
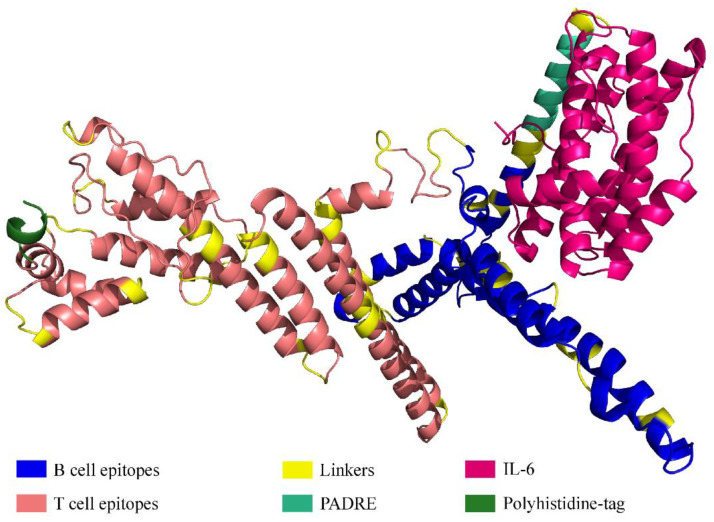
Tertiary structure of the protein comprising multiple epitopes.

**Figure 5:**
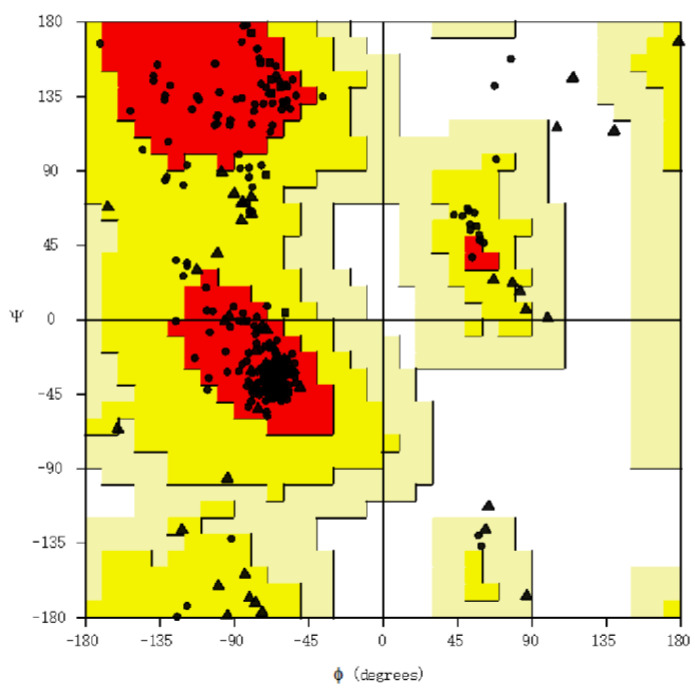
Ramachandran plot assessing vaccine structure quality. Each dot shows the amino acids. The positioning of these dots reflects the backbone dihedral angles ψ versus φ of the amino acids present in the vaccine product. Triangles indicate glycine, squares indicate prolines, while circles denote non-glycine and non-proline residues.

Using RoseTTAFold, the tertiary structure of the protein of the vaccine was predicted and subsequently refined by GalaxyRefine (Figure 4). The tool generated five models out of which the one with the better characteristics was selected for the docking analysis. Of the amino acids, 93.0% were located in the most favored region of the Ramachandran, 6.3% were in the allowed region, 0.4% were in regions that were generously allowed, and 0.4% of the amino acid residues were in the outlier region. These parameters, along with the ERRAT with an overall quality factor of 92.9825, indicate the desired level of the quality of the vaccine product (Figure 5). 

### 3.3. Physicochemical Properties, Allergenicity, and Antigenicity Analyses

According to the physicochemical properties predicted by ProtParam, the vaccine sequence contains 647 amino acid residues, and its MW is 68.97777 kDa. The theoretical pI was computed to be 9.31. The vaccine contains 55 negatively charged residues (Asp + Glu). The instability index was 39.82, which classifies the vaccine as stable. The estimated half-life in vitro was 100 h, while the estimated half-lives in vivo in yeast and *E. coli* were 20 h and 10 h, respectively. The aliphatic index was 82.32, which implies that the vaccine construct has a high level of thermostability. The vaccine’s GRAVY score was −0.233, which suggests that the protein is hydrophilic.

The allergenicity prediction via the AlgPred server showed that the multi-epitope vaccine was non-allergen, while the server VaxiJen v2.0 with the default parameters and the threshold 0.4 [54] demonstrated its probable antigenicity with the antigenic score 0.4656.

### 3.4. Conformational B Cell Epitopes

**Figure 6:**
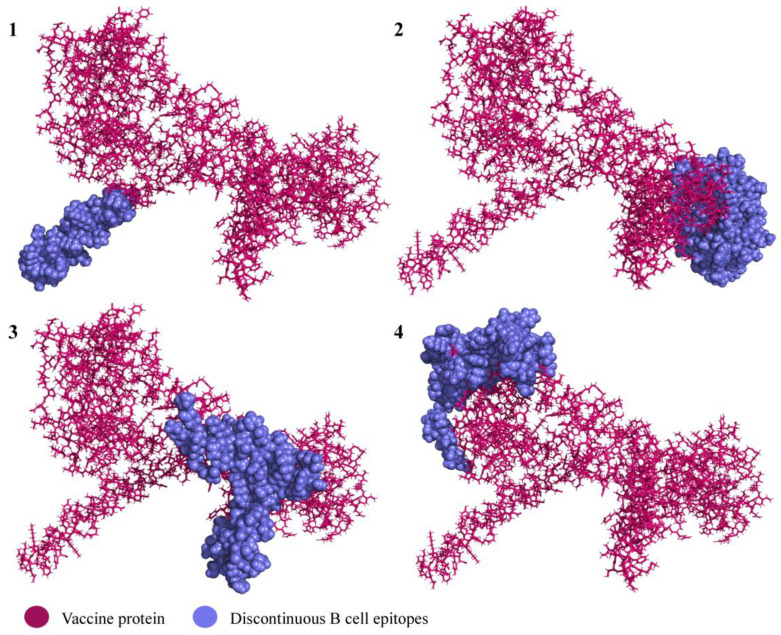
Predicted discontinuous epitopes of B cell epitopes in the vaccine product. The subfigure numbers (**1**–**4**) correspond to the conformational B cell epitope numbers listed in Table 2.

In total, four discontinuous B cell epitopes were predicted with the scores 0.69–0.859. The sizes varied between 44 and 109 residues. The presence of the conformational epitopes in the vaccine product indicates its potential capacity to induce a humoral immune response when the vaccine is administered in vivo. All the predicted discontinuous epitopes are given in Table 2 and illustrated in Figure 6. 

### 3.5. Molecular Docking and Immune Responses following Immune Simulation

**Figure 7:**
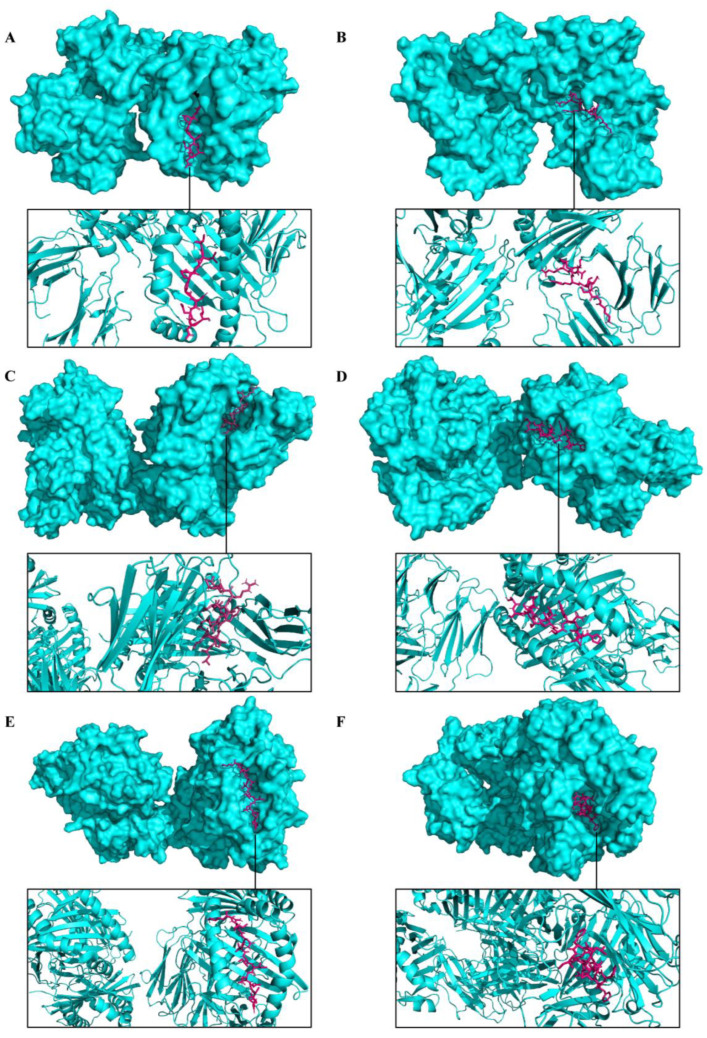
Representation of predicted molecular docking between epitopes of a multi-epitope vaccine construct and host receptors. The MHC molecules are displayed in cyan color and the epitope is shown in magenta. (**A**) Docked complex of epitope TLFPGDDDL (A29/A30/A27) and MHC-I. (**B**) Docked complex of epitope NTLSERISSK (M1/M1/L1) and MHC-I. (**C**) Docked complex of epitope LSMITMSAFLIVRLN (A35/A36/A33) and MHC-II. (**D**) Docked complex of epitope ASYISCTANSWNVIP (B6/B7/B5) and MHC-II. (**E**) Docked complex of epitope KIQNVIIDECY (M1/M1/L1) and MHC-II. (**F**) Docked complex of epitope FFIVVATAAVCLLFI (A30/A31/A28) and MHC-II.

**Figure 8:**
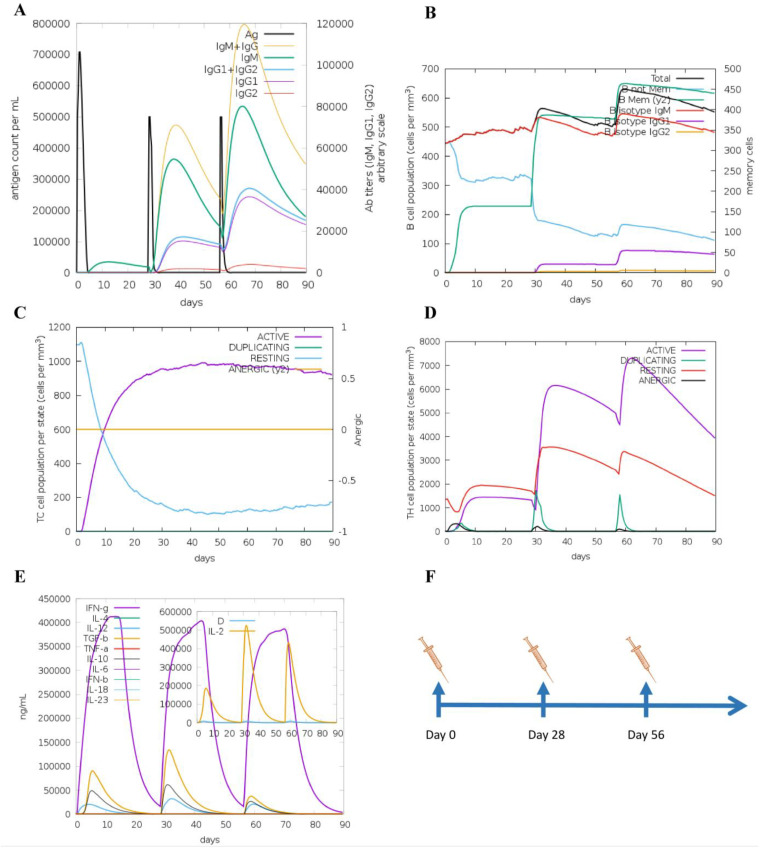
Immune responses elicited after in silico immunization. (**A**) Antibodies produced in response to immune simulation. Immunoglobulin subclasses are given in different colors. (**B**) Representation of evolution of B cell populations following the immunization with three doses. (**C**) Generation of CD8 T-cytotoxic lymphocytes induced by antigen exposure. The resting state denotes T-cytotoxic cells not actively engaged in fighting the infection, while the anergic state denotes the unresponsiveness of the CD8 T-cytotoxic cells due to repeated and prolonged exposure to the antigen. (**D**) Production of CD4 T-helper cells induced by exposure to the vaccine antigen. (**E**) The cytokine profile in response to the vaccine injections. The inserted graph shows the IL-2 level with the Simpson Index. D indicates a danger signal. (**F**) Schedule of in silico immunization.

The molecular docking analysis of epitopes with the MHC-I and MHC-II molecules revealed the stable binding (Figure 7). The molecular docking results are given in the Supplementary Materials (Tables S11 and S12). In total, three doses were administered four weeks apart for the immune simulation—day 0, day 28, and day 56 with an 8 h offset. As expected, the immune response was also compatible with the immune responses that are elicited in general after in vivo immunization. The immune responses after receiving additional and booster doses were more robust compared to the first shot. The immune responses following the second and third shots were stronger in comparison to the prime immunization. The antigen level was decreased while elevated levels of antibodies, including IgG1, IgG1 + IgG2, IgM, and IgG + IgM, were detected. Antigen abundance peaked at each vaccine injection (Figure 8A). The humoral response surged after each immunization, and the antibody levels remained high for weeks after the last vaccine injection. Following immunization, the activation of the B cell population, resulting in an increased number of B cells producing antibodies, was observed (Figure 8B). The counts of CD8 T-cytotoxic lymphocytes show the ignorable number of anergic cells and activation of T-cytotoxic cells (Figure 8C). The counts of CD4 T-helper lymphocytes demonstrate that the duplication phase starts immediately after each injection (Figure 8D). The cytokine production was also manifested upon immunization; particularly, high levels of IFN-γ, TGF-β, and IL-10 were induced by the infection (Figure 8E). The other parameters of the immune response elicited by the vaccine are presented in the Supplementary Materials (Figure S1).

## 4. Discussion

Mpox was known to be a rare zoonotic disease caused by the MPXV that usually emerged in rural rainforest regions of African countries [7]. The genus *Orthopoxvirus* contains four species that are pathogenic to humans: VARV (the causative agent of smallpox), MPXV, VACV, and cowpox. Smallpox and mpox are often life-threatening diseases, whereas vaccinia and cowpox are generally associated with local lesions. MPXV received this name as it was first isolated from laboratory macaque monkeys imported from Singapore to Copenhagen, Denmark, in 1958 [55]; however, it is considered that the host and the main source of MPXV are rodents such as squirrels, Gambian rats, etc. [56]. The first human case was documented in the Democratic Republic of the Congo (DRC) in 1970 while smallpox surveillance was taking place [7]. Particularly, a nine-month-old boy was admitted to the hospital with suspected smallpox. The specimens were sent to the Smallpox Reference Centre of the World Health Organization (WHO) where MPXV was isolated [57]. The symptoms of mpox include fever, malaise, respiratory symptoms, fatigue, lymphadenopathy, headache, and muscle aches, along with the main symptom—a rash similar to pimples and blisters that are itchy and painful and eventually crust over and are healed. The manifestation of mpox is similar to other rash diseases, hence, misdiagnoses often take place. Instead of mpox, mostly, chickenpox caused by *Varicella zoster* virus is mistakenly diagnosed [15,58,59]. The rush is usually concentrated on the face and extremities including palms and soles of the feet; however, according to the evidence during the current mpox outbreak, it is also localized in the perineal/perianal area as well as on the genitals [60,61]. Although mpox usually resolves by itself, the recent 2022 outbreak demonstrated that it is a life-threatening disease [62]. Indeed, after all, MPXV is categorized as a high-threat virus that belongs to biosafety level 3, according to EU regulations [63]. The possible reasons why MPXV is being spread more than usual include the increased exotic animal trade and international travel [58], the introduction from a single origin with super-spreader events [64], the long period of MPXV cryptic dissemination in humans as well as in animals in non-endemic countries [64], the affected human immunity levels due to the COVID-19 global pandemic while gaining the adaptability by the MPXV, and the waning of the immunity against smallpox in the world population which is caused by terminating the vaccination since 1980 when the disease was announced eradicated by WHO [7,55,56,65]. The latter one seems more reasonable as today only approximately 30% of the world population is vaccinated against smallpox [58] and the majority of infected people are younger than 50 and have never been vaccinated against smallpox. Vaccination to smallpox induced coincident immunity to MPXV, but smallpox eradication and halted vaccination as well as lack of vaccination triggered the MPXV to acquire clinical relevance [66]. Although the smallpox vaccine protects from MPXV [67], there are cases of MPXV infection in patients who are vaccinated against smallpox [18]. Mpox has the potential to grow as a global threat which necessitates the development of a specific vaccine. Moreover, if MPXV is spread easily due to the waned immunity against smallpox, smallpox itself might also re-emerge anytime soon, and that could be a huge plague. In addition, the “forgotten” viruses should get more attention as from time to time they manage to emerge, and in the case of the highly deadly virus, it would cause an unprecedentedly adverse outcome for the human population, e.g., Crimean-Congo hemorrhagic fever (CCHF) virus, Zika, Ebola, etc. Although they are considered to be limited geographically, the possibility of their spreading wider is high. MPXV was also reckoned as geographically limited and did not get much attention; however, in 2003, the outbreak took place outside of Africa for the first time which led to the increased attention to this virus. Moreover, there is a number of cases that are not reported due to the lack of surveillance systems in endemic areas, which most possibly resulted in the expansion of geographic areas [68]. According to the history of epidemics and pandemics, although the number of cases is not increasing recently, the new wave of MPXV can pose a higher risk to the health of the world population. Hence, it is important to design a more specific and universal vaccine against MPXV that will have fewer side effects. As MPXV, VARV, and VACV are from the same genus and their genomes are conserved, it is prudent to develop a vaccine based on the conserved elements. In this study, five antigens—A29, A30, A35, M1, and B6—were selected according to their importance for the viral life cycle. Importantly, these antigens have shown favorable outcomes when they were used in the line of studies and elicited protection from the MPXV challenge. Buchman et al., as well as Heraud et al., have challenged the non-human primates intradermally with the lethal dose of MPXV after the vaccination with a subunit vaccine consisting of VACV membrane proteins A33, B5, L1, and A27 [28,29] (see the names of the corresponding proteins of MPXV and VARV in Table 1), and all the animals survived. Hooper et al. used a DNA vaccine comprising the same antigens of VACV and demonstrated that the immunization with DNA vaccine encoding these four antigens protected rhesus macaques from a severe disease after the lethal MPXV challenge [27]. On the other hand, Hirao et al. used a DNA vaccine encoding eight antigens of VACV—A4, A56, F9, H3, A27, A33, B5, and L1—to immunize cynomolgus macaques and showed that the vaccination elicited protective immunity in the animals [24]. As to the fifth selected antigen in the current study, A28, it is one of at least eight transmembrane proteins in the VACV entry/fusion complex subunit and is evidenced to be a target of neutralizing and protective antibodies in rabbits [69]. 

The vaccine used for the eradication of smallpox was the live VACV-based vaccine that necessitated special precautions to protect from spreading the VACV from their vaccination spot [70]. Although the current VACV-based vaccine that is currently used against MPXV is a highly effective attenuated Modified Vaccinia Ankara-Bavarian Nordic (MVA-BN) virus [71,72], the development of a potentially universal next-generation vaccine against MPXV, VARV, and similar viruses from the same genus is reasonable. Nucleic acid vaccines, specifically mRNA-based vaccines, have recently garnered significant interest due to their remarkable advantages over other types of nucleic acid and conventional vaccines [73]. mRNA vaccines are highly potent, safe, cost-effective, and rapidly manufactured [19,20,21]. Indeed, the current COVID-19 pandemic has proven the superiority of mRNA vaccines over any other vaccines [74,75,76]. The advantages of mRNA-based immunization include efficacy, safety (no risk of genome integration as it is directly translated into the cytoplasm unlike DNA vaccines), cell-free manufacturing, and fast production. Hence, as mRNA technology represents a promising strategy, designing an mRNA vaccine against MPXV, VARV, and VACV is definitely reasonable [77,78].

Smallpox is the only infectious disease that is considered to be globally eradicated since 1980 [79]. However, the course of pandemic events in world history has revealed that a new pandemic can emerge unexpectedly despite the rapid advancement of science with numerous valuable studies. Thus, even though smallpox has been eliminated, there is a risk of re-emergence. Moreover, in the past, it killed millions of people until the effective vaccine was developed [80]. The current outbreak of mpox is an example of it. In addition, although VACV is not regarded as dangerous, evidently, it causes infection [81], and there is a possibility that it will become more virulent in the future. Accordingly, designing a potentially universal vaccine that can be protective to MPXV as a current emergency, VARV as a potential re-emergency, and VACV as its effective application as a vaccine seems prudent. 

Noteworthily, a number of excellent immunoinformatics tools are available for finding the conserved sequences of the selected antigens [82,83,84], for finding experimentally tested epitopes or predicting the epitopes on the IEDB [31], and for the prediction of certain properties and immune response induced by the designed vaccine [30,40,41,42,43,44,45,46,47,49,51,85,86,87]. These in silico analyses save time and allow us to predict the potential outcome of the designed vaccine. Moreover, rather than commencing the time-consuming and expensive in vitro/in vivo experiments directly, using the immunoinformatics analysis that is favorable to formulate the universal MPXV mRNA vaccine and screen the multi-epitope construct containing conserved elements using in silico approaches seems very reasonable. Indeed, a line of research has been dedicated to developing vaccines for COVID-19 [41,88], influenza [89,90,91], and other viruses [92,93,94] using bioinformatics approaches. Until now, there is no study focusing on designing a multi-epitope universal mRNA vaccine against MPXV, VARV, and VACV together. Hence, in the current study, epitopes of T cells that have already undergone experimental testing were chosen for their potential to enhance the favorable results of the proposed mRNA vaccine. On the other hand, B cell epitopes were predicted via IEDB prediction software [32] as there were no experimentally tested B cell epitopes found in the database. As the optimization of the final construct is of great importance and linkers that connect the epitopes to each other play a crucial role, flexible KK [41] and GGGS [35] linkers along with a rigid EAAAK linker [34,38] were used. The flexible linkers used here improved folding and stability in fusion proteins, while the EAAAK attached the peptides to each other to achieve an immunologically active multi-peptide [33].

In silico immunization showed that the vaccine designed for MPXV, VARV, and VACV in this study elicits optimal humoral and cellular immune responses following repeated immunization. In silico immunization with the proposed vaccine stimulated the B cell population, and increased the production of immunoglobulins, CD8 T-cytotoxic and CD4 T-helper cells, memory cells, as well as cytokines. In addition, the molecular docking showed favorable binding to the MHC molecules and other physicochemical parameters of the vaccine demonstrated stability and optimal characteristics of the vaccine. 

**Figure 9:**
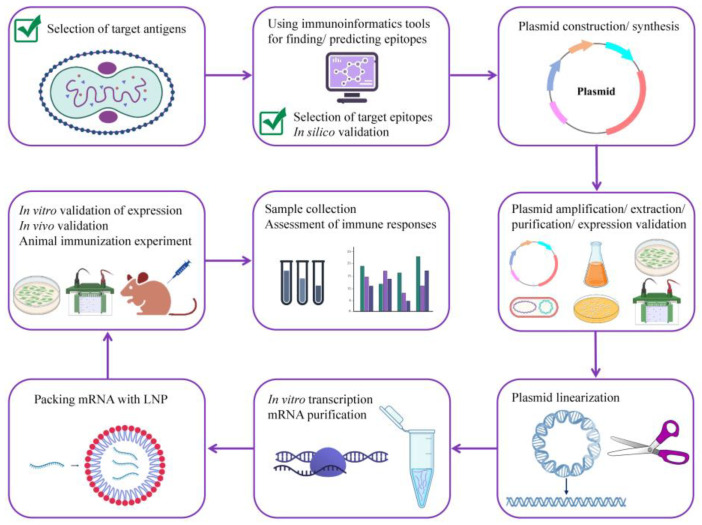
A roadmap for developing a potentially universal multi-epitope mRNA vaccine candidate against MPXV. MPXV, monkeypox virus.

The in vivo validation of the efficacy and safety of the potentially universal mRNA vaccine, which was designed and tested in silico in this study, is unquestionably necessary. For this, the multi-epitope antigen should be subcloned into an expression vector, transformed into DH5α competent cells, amplified, extracted and purified, linearized, in vitro transcribed, and encapsulated with lipid nanoparticles (LNPs). Then, the animal immunization experiment should be conducted for the assessment of immune responses. The schematic illustration of the steps needed for the development of a potentially universal mRNA vaccine candidate for MPXV, VARV, and VACV is shown in Figure 9.

## 5. Conclusions

Taken together, it is conceivable that mpox outbreaks will take place more frequently in the future [95]. Thus, more research is urgently needed for developing a specific and universal MPXV vaccine and therapeutics for the prevention and treatment of the disease, respectively. To the best of our knowledge, this is the first study focusing on designing of multi-epitope universal mRNA MPXV vaccine that will be potentially effective against VARV and VACV as well. Indeed, immunoinformatics presents a promising strategy for contributing to the rapid development of mRNA vaccines, which could be achieved through subsequent pre-clinical and clinical investigations. This would enable the prevention, mitigation, and management of potential outbreaks before they escalate into deadly pandemics. Moreover, given that predicting the timing and location of the next pandemic is unfeasible, it is essential to have readily developed vaccine candidates that can be used swiftly to contain outbreaks before they escalate into worldwide pandemics. In summary, the current study demonstrated that the multi-epitope construct for mpox proposed here seems to be an auspicious candidate owing to the potential to induce an immune response against the two clades of MPXV and same family viruses—VACV and VARV—that might lay the groundwork to the alertness for epidemics and pandemics.

## Figures and Tables

**Table 1:** Selected antigens among MPXV, VARV, and VACV, and their location in the virus, function, and characteristics.

Name (MPXV)	Name (VARV)	Name (VACV)	Location	Function and Characteristics
A29	A30	A27	MV	Surface membrane fusion protein; Binds to cell surface heparan; Neutralizing antibody target
A30	A31	A28	MV	Envelope protein; Virus entry into a host; Cell–cell fusion (syncytial formation); Neutralizing antibody target
A35	A36	A33	EV	Envelope glycoprotein; Formation of actin-containing microvilli and cell-to-cell spread of virion; Neutralizing antibody target; Target of complement-mediated cytolysis
B6	B7	B5	EV	Palmitylated glycoprotein; Required for efficient cell spread; Complement control
M1	M1	L1	MV	Myristylated surface membrane protein; Virus entry into a host; Neutralizing antibody target

Notes: MPXV, monkeypox virus; VARV, variola virus; VACV, vaccinia virus.

**Table 2:** Discontinuous B cell epitopes predicted by ElliPro.

#	Epitopes	Residue Number	Score
1	A:K287, A:E289, A:Q290, A:T291, A:S292, A:K293, A:K294, A:V295, A:S296, A:E297, A:L298, A:Y299, A:N300, A:K301, A:P302, A:L303, A:Y304, A:K305, A:K306, A:E307, A:E308, A:K309, A:N310, A:G311, A:N312, A:T313, A:S314, A:W315, A:N316, A:D317, A:T318, A:V319, A:K320, A:K321, A:P322, A:D323, A:D324, A:E325, A:T326, A:D327, A:L328, A:S329, A:K330, A:L331	44	0.859
2	A:I511, A:V512, A:R513, A:L514, A:N515, A:Q516, A:C517, A:M518, A:S519, A:A520, A:N521, A:G522, A:G523, A:G524, A:S525, A:A526, A:S527, A:Y528, A:I529, A:S530, A:C531, A:T532, A:A533, A:N534, A:S535, A:N537, A:I539, A:G546, A:V547, A:I548, A:H549, A:L550, A:S551, A:C552, A:K553, A:S554, A:G555, A:F556, A:I557, A:L558, A:T559, A:G560, A:G561, A:G562, A:S563, A:M564, A:K565, A:T566, A:I567, A:S568, A:V569, A:V570, A:S589, A:V592, A:L593, A:V594, A:C595, A:S596, A:C597, A:N598, A:G599, A:G600, A:G601, A:S602, A:K603, A:I604, A:Q605, A:N606, A:V607, A:I608, A:I609, A:D610, A:E611, A:C612, A:Y613, A:G614, A:G615, A:G616, A:S617, A:A618, A:A619, A:L620, A:F621, A:M622, A:Y623, A:Y624, A:A625, A:K626, A:R627, A:G628, A:G629, A:G630, A:S631, A:N632, A:T633, A:L634, A:S635, A:E636, A:R637, A:I638, A:S639, A:S640, A:K641, A:H642, A:H643, A:H644, A:H645, A:H646, A:H647	109	0.761
3	A:K212, A:D216, A:N217, A:K218, A:K221, A:G224, A:G225, A:T226, A:P227, A:A228, A:K229, A:K230, A:Q231, A:D232, A:V233, A:N234, A:D235, A:T236, A:I237, A:S238, A:D239, A:K240, A:K241, A:G242, A:P243, A:N244, A:N245, A:T246, A:R247, A:K248, A:K249, A:S250, A:T251, A:H252, A:R253, A:K254, A:V255, A:F401, A:I402, A:V403, A:V404, A:A405, A:T406, A:A407, A:A408, A:V409, A:C410, A:L411, A:L412, A:F413, A:I414, A:G415, A:G416, A:G417, A:S418, A:M419, A:N420, A:S421, A:L422, A:S423, A:I424, A:F425, A:F426, A:I427, A:V428, A:V429, A:A430, A:T431, A:A432, A:A433	70	0.733
4	A:D9, A:V10, A:A11, A:A12, A:P13, A:H14, A:R15, A:Q16, A:P17, A:L18, A:T19, A:S20, A:S21, A:E22, A:R23, A:I24, A:D25, A:M66, A:A67, A:E68, A:K69, A:D70, A:G71, A:C72, A:F73, A:Q74, A:S75, A:G76, A:F77, A:N78, A:E79, A:E80, A:T81, A:C82, A:L83, A:V84, A:K85, A:I86, A:I87, A:T88, A:L91, A:V120, A:L121, A:I122, A:Q123, A:F124, A:L125, A:Q126, A:K128, A:A129, A:K130, A:N131, A:L132, A:D133, A:A134, A:I135, A:T136, A:T137, A:P138, A:D139, A:P140, A:T141, A:T142, A:A144, A:S145, A:T148, A:K149, A:S176, A:L177, A:R178, A:A179, A:L180, A:R181, A:Q182, A:M183, A:G184, A:G185, A:G186, A:S187, A:A188, A:K189, A:F190, A:V191, A:A192, A:A193, A:W194, A:T195, A:K197, A:A198, A:E201	90	0.69

## Data Availability

The datasets analyzed for this study can be found in the NCBI database [https://www.ncbi.nlm.nih.gov/; accessed on 19 August 2022] and the Immune Epitope Database (IEDB) [https://www.iedb.org/; accessed on 19 August 2022]. The data that support the findings of this study are available from the corresponding authors upon reasonable request. All data created during this study are included in this article. Further inquiries can be directed to the corresponding authors.

## Supplementary Materials

The following supporting information can be downloaded at: https://www.mdpi.com/article/10.3390/v15051120/s1, Figure S1: Immune simulation results; Table S1: List of T cell epitopes (antigen A29L-A30L-A27L) containing conserved amino acid sequences (origin VACV); Table S2: List of B cell epitopes (antigen A29L-A30L-A27L); Table S3: List of T cell epitopes (antigen A30L/A31L/A28L) containing conserved amino acid sequences (origin VACV); Table S4: List of B cell epitopes (antigen A30L/A31L/A28L); Table S5: List of T cell epitopes (antigen A35R-A36R-A33R) containing conserved amino acid sequences (origin VACV); Table S6: List of B cell epitopes (antigen A35R-A36R-A33R); Table S7: List of T cell epitopes (antigen B6R-B7R-B5R) containing conserved amino acid sequences (origin VACV and VARV); Table S8: List of B cell epitopes (antigen B6R-B7R-B5R); Table S9: List of T cell epitopes (antigen M1R-M1R-L1R) containing conserved amino acid sequences (origin VACV); Table S10: List of B cell epitopes (antigen M1R-M1R-L1R); Table S11: Molecular docking results of the final vaccine constructs with MHC-I; Table S12: Molecular docking results of the final vaccine constructs with MHC-II.
