# Supplementary material for: Development of a Multi-Epitope Universal mRNA Vaccine Candidate for Monkeypox, Smallpox, and Vaccinia Viruses: Design and In Silico Analyses
Source: Viruses. 2023 May 7;15(5):1120. doi: 10.3390/v15051120 (PMC10222975; doi:10.3390/v15051120)
Supplement: Supplementary file 1 [file viruses-15-01120-s001.zip › viruses-2373988-supplementary.pdf]

Supplementary materials

Open reading frame amino acid sequence of monkeypox multi-epitope mRNA vaccine construct

MDAMKRGLCCVLLLCGAVFVSPSVPPGEDSKDVAAPHRQPLTSSERIDKQIRYILDGISALRKETCNKS
NMCESSKEALAENNLNLPKMAEKDGCFQSGFNEETCLVKIITGLLEFEVYLEYLQNRFESSEEQARAV
QMSTKVLIQFLQKKAKNLDAITTPDPTTNASLLTKLQAQNQWLQDMTTHLILRSFKEFLQSSLRALRQ
MGGGSAKFVAAWTLKAAAEAAAKVQTGRKKDGDDNKKSKSIGGTPAKKQDVNDTISDKKGPNNTR
KKSTHRKVASSTTQYDHKKKPITKTTSDYQDSDVSQEVKKEEQTSKKVSELYNKPLYKKEEKNGNTS
WNDTVKKPDDETDLSKLSKDKKEANASKKQTSVNTKKAPRQVGGGSTLFPGDDDLGGGSTLKQRLT
NLGGGSTLRAAMISLGGGSFFIVVATAAVCLLFIGGGSMNSLSIFFIVVATAAVCLLFIGGGSATAAVCLL
FIQGYSIYENYGNGGGSLSMITMSAFLIVRLNGGGSRVIGLCIRISMVISLGGGSSAFLIVRLNQCMSAN
GGGSASYISCTANSWNVIPGGGSGGVIHLSCKSGFILTGGGSMKTISVVTLLCVLPAGGGSGVIFLISVI
VLVCSCNGGGSKIQNVIIDECYGGGSAALFMYYAKRGGGSNTLSERISSKHHHHHH

**Signal peptide of TPA**	MDAMKRGLCCVLLLCGAVFVSPS
**IL-6**	VPPGEDSKDVAAPHRQPLTSSERIDKQIRYILDGISALRKETCNKSNMC ESSKEALAENNLNLPKMAEKDGCFQSGFNEETCLVKIITGLLEFEVYL EYLQNRFESSEEQARAVQMSTKVLIQFLQKKAKNLDAITTPDPTTNAS LLTKLQAQNQWLQDMTTHLILRSFKEFLQSSLRALRQM
**PADRE**	AKFVAAWTLKAAA

Conserved sequences of antigens which were used to find and predict corresponding epitopes

A29L/A30L/A27L:
MDGTLFPGDDDLAIPATEFFST;
KREAIVKA;
DDNEETLKQRLTNLEKKITN;
TTKFEQIEKCCK;
NDEVLFRLENHAETLRAAMISLAKKIDVQTGR.

A30L/A31L/A28L:
MNSLSIFFIVVATAAVCLLFIQ;
YSIYENYGNIKEFNATHAAFEYSKSIGGTPALDRRVQDVND;
ISDVKQKWRCV;
YPGNGFVSASIFGFQAEVGPNNTRSIRKFNTM;
QCIDFTFSDVINI;
IYNPC;
PNINN;
ECQFLKSVL.

A35R/A36R/A33R:
MMTPENDEEQTSVFSATVY;
DKIQGKNKRKRVIGLCIRISMVISLLSMITMSAFLIVRLNQCMSAN;
AAITD.

B6R/B7R/B5R:
MKTISVVTLLCVLPAVVYSTCTVPTMNNAKLTSTETSFN;
QKVTFTCD;
DPNAVCETDKWKYENPCKKMCTVSDY;
KPLYEVNSTMTLSCNGETKYFRC;
EKNGNTSWNDTVTCPNAECQPLQLEHGSCQPVKEKYSFGEY;
TINCDVGYEVIG;
YISCTANSWNVIPSCQQKCD;
PSLSNGLISGSTFSIGGVIHLSCKSGF;
LTGSPSSTCIDGKWNP;
FDPVDDGPDDETDLSKLSKDVVQYEQEIESLEATYHIII;
ALTIMGVIFLIS;
IVLVCSCDKNNDQYKFHKLLL.

M1R/M1R/L1R:
MGAAASIQTTVNTLSERISSKLEQEANASAQTKCDIEIGNFYIRQNHGCNITVKNMCSADADAQLDAVLSAATE
TYSGLTPEQKAYVPAMFTAALNIQTSVNTVVRDFENYVKQTCNSSAVVDNKLKIQNVIIDECYGAPGSPTNLEF
INTGSSKGNCAIKALMQLTTKATTQIAP;
QVAGTGVQFYMIVIGVIILAALFMYYAKRMLFTSTNDKIKLILANKENVHWTTYMDTFFRTSPM;
IIATTD

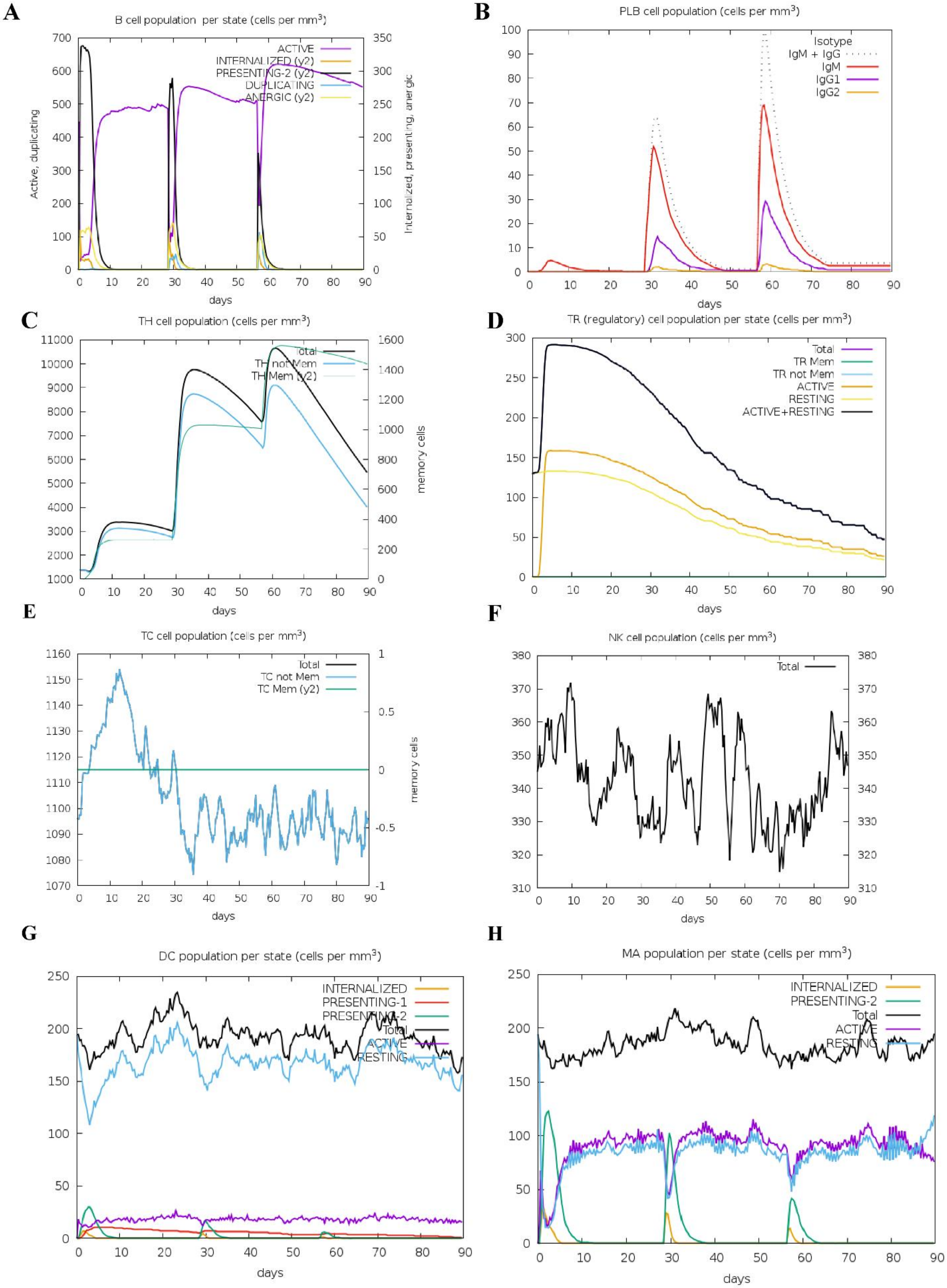
A
B cell population per state (cells per mm3)
ACTIVE
INTERNALIZED (y2)
PRESENTING-2 (y2)
DUPLICATING
ANERGIC (y2)
Active, duplicating
Internalized, presenting, anergic
days
B
PLB cell population (cells per mm3)
Isotype
IgM + IgG
IgM
IgG1
IgG2
days
C
TH cell population (cells per mm3)
Total
TH not Mem
TH Mem (y2)
memory cells
days
D
TR (regulatory) cell population per state (cells per mm3)
Total
TR Mem
TR not Mem
ACTIVE
RESTING
ACTIVE+RESTING
days
E
TC cell population (cells per mm3)
Total
TC not Mem
TC Mem (y2)
memory cells
days
F
NK cell population (cells per mm3)
Total
days
G
DC population per state (cells per mm3)
INTERNALIZED
PRESENTING-1
PRESENTING-2
Total
ACTIVE
RESTING
days
H
MA population per state (cells per mm3)
INTERNALIZED
PRESENTING-2
Total
ACTIVE
RESTING
days

Figure S1. Immune simulation results. (A) B cell population per entity-state showing counts of active, presenting, duplicating cells, etc. (B) Plasma B lymphocytes count sub-divided per isotype (IgM, IgG1 and IgG2). (C) CD4 T-helper lymphocytes count. The plot shows total and memory counts. (D) Count of CD4 T-regulatory lymphocytes (total, memory, and per entity-state counts). (E) Count of CD8 T-cytotoxic lymphocytes. (F) Total count of Natural Killer cells. (G) Total count of active, resting, internalized, and antigen-presenting dendritic cells. (H) Total count of internalized, presenting on MHC class-II, active, and resting macrophages.

Supplementary tables

Table S1. List of T cell epitopes (antigen A29L-A30L-A27L**)** containing conserved amino acid sequences (origin VACV)

#	Epitope	Epitope ID	Length	Position	Publications/Assays	Summary
1	AKKPEAKREAIVKAD	2216	15	25-39	Tested in 1 T Cell Assay	IFNγ release – 1/1
2	ETLKQRLTNLEKKIT	14458	15	45-59	Tested in 2 T Cell Assay	IFNγ release – 2/2
3	EVLFRLENHAETLRA	14762	15	77-91	Tested in 1 T Cell Assay	IFNγ release – 1/1
4	KQRLTNLEK	33061	9	48-56	3 publications, tested in 5 T cell assays and 1 MHC ligand assay	T cell assays:<br>IFNγ release – 1/5<br>MHC ligand assays:<br>HLA-A*30:01 – 1/1
5	LRAAMISLAKKIDVQ	39027	15	89-103	3 publications, tested in 3 T cell assays and 1 MHC ligand assay	T cell assays:<br>Cytotoxicity – 2/2<br>IFNγ release – 0/1<br>MHC ligand assays:<br>H2-IAb – 1/1
6	TLFPGDDDL	64800	9	4-12	4 publications, tested in 3 T cell assays and 1 MHC ligand assay	T cell assays:<br>IFNγ release – 2/3<br>MHC ligand assays:<br>HLA-A*02:01 – 1/1
7	TLKQRLTNL	64883	9	46-54	6 publications, tested in 3 T cell assays and 5 MHC ligand assays	T cell assays:<br>IFNg release – 1/3<br>MHC ligand assays:<br>HLA-A*02:01 – 4/4<br>HLA-B*08:01 – 1/1
8	TLRAAMISL	65031	9	88-96	6 publications, tested in 3 T cell assays and 3 MHC ligand assays	T cell assays:<br>IFNγ release – 1/3<br>MHC ligand assays:<br>HLA-A*02:01 – 2/2<br>HLA-A*30:01 – 1/1
9	AAMISLAKK	80142	9	91-99	Tested in 1 MHC ligand assay	HLA-A*30:01 – 1/1
10	LAIPATEFF	84958	9	12-20	Tested in 1 MHC ligand assay	HLA-B*15:01 – 1/1
11	EVLFRLENHAETLRAA	140023	16	77-92	Tested in 1 T Cell Assay	IFNγ release – 1/1

12	KADEDDNEETLKQRLT	140040	16	37-52	Tested in 1 T Cell Assay	IFNγ release – 1/1
13	KITNVTTKFEQIEKCC	140046	16	57-72	Tested in 1 T Cell Assay	IFNγ release – 1/1
14	KRNDEVLFR	1625401	9	73-81	Tested in 1 MHC ligand assay	HLA-B*27:05 – 1/1

Table S2. List of B cell epitopes (antigen A29L-A30L-A27L)

A29L-A30L-A27L (MPXV, VARV, and VACV) – Consensus sequence	Predicted linear epitopes	Position
>Consensus/1-110 Percentage Identity Consensus<br>MDGTLFPGDDDLAIPATEFFSTKAAKKPEAKREAIVKADGDDNEE<br>TLKQRLTNLEKKITNVTTKFEQIEKCCKRNDEVLFRLENHAETLR<br>AAMISLAKKIDVQTGRRPYE	DGDDN	38-42
	VQTGR	101-105

Table S3. List of T cell epitopes (antigen A30L/A31L/A28L**)** containing conserved amino acid sequences (origin VACV)

#	Epitope	Epitope ID	Length	Position	Publications/Assays	Summary
1	ATAAVCLLFIQGYSIYENYGN	4868	21	12-32	Tested in 2 T cell assays and 1 MHC ligand assay	T cell assays:<br>IFNγ release – 2/2<br>MHC Ligand Assays:<br>HLA-DRB1*01:01 – 1/1
2	FFIVVATAAVCLLFI	15833	15	7-21	3 publications, tested in 2 T cell assays and 3 MHC ligand assays	T cell assays:<br>IFNγ release – 2/2<br>MHC ligand assays:<br>H2-IAb – 2/2<br>HLA-DRB1*04:01 – 1/1
3	FIVVATAAV	16327	9	8-16	6 publications, tested in 6 T cell assays and 3 MHC ligand assays	T cell assays:<br>IFNγ release – 2/6<br>MHC Ligand Assays:<br>HLA-A*02:01 – 3/3
4	MNSLSIFFIVVATAAVCLLFI	42240	21	1-21	Tested in 2 T cell assays and 1 MHC ligand assay	T cell assays:<br>IFNγ release – 2/2<br>MHC ligand assays:<br>HLA-DRB1*01:01 – 1/1

5	RSIRKFNTM	55865	9	101-109	2 publications, tested in 2 T cell assays and 2 MHC ligand assays	T cell assays: IFNγ release – 0/2 MHC ligand assays: HLA-B*08:01 – 1/1 HLA-B*15:01 – 1/1
6	SIFGFQAEV	58497	9	87-95	4 publications, tested in 2 T cell assays and 2 MHC ligand assays	T cell assays: IFNγ release – 0/2 MHC ligand assays: HLA-A*02:01 – 2/2
7	LSIFFIVVA	85829	9	4-12	Tested in 1 MHC ligand assay	HLA-B*15:01 – 1/1
8	NINNAECQF	86779	9	133-141	Tested in 1 MHC ligand assay	HLA-B*15:01 – 1/1
9	AVCLLFIQGYSIYEN	190204	15	15-29	Tested in 2 MHC ligand assays	HLA-DRB1*01:01 – 2/2

Table S4. List of B cell epitopes (antigen A30L/A31L/A28L)

A30L-A31L-A28L (MPXV, VARV, and VACV) – Consensus sequence	Predicted linear epitopes	Position
>Consensus/1-146 Percentage Identity Consensus MNSLSIFFIVVATAAVCLLFIQSYSIYENYGNIKEFNATHAAFEY SKSIGGTPALDRRVQDVNDTISDVKQKWRCVVYPGNGFVSASI FGFQAEVGPNNTRSIRKFNTMRQCIDFTFSDVINIDIYNPCIAPNI NNTECQFLKSVL	SKSIGGTPA	45-53
	QDVNDTISD	59-67
	GPNNTR	95-100

Table S5. List of T cell epitopes (antigen A35R-A36R-A33R**)** containing conserved amino acid sequences (origin VACV)

#	Epitope	Epitope ID	Length	Position	Publications/ Assays	Summary
1	CMSANEAAI	6667	9	62-70	3 publications, tested in 1 T cell assay and 2 MHC ligand assays	T cell assays: IFNγ release – 0/1 MHC ligand assays: HLA-A*02:01 – 2/2
2	GLYYQGSCYI	21244	10	102-111	3 publications, tested in 1 T cell assay and 2 MHC ligand assays	T cell assays: IFNγ release – 0/1 MHC ligand assays: HLA-A*02:01 – 2/2

3	ITMSAFLIV	28998	9	49-57	6 publications, tested in 3 T cell assays and 4 MHC ligand assays	T cell assays:<br>IFNγ release – 0/3<br>MHC ligand assays:<br>HLA-A*02:01 – 2/2<br>Mamu-A1*001:01 – 2/2
4	LITWLIDYV	36737	9	140-148	4 publications, tested in 2 T cell assays and 2 MHC ligand assays	T cell assays:<br>IFNγ release – 0/2<br>MHC ligand assays:<br>HLA-A*02:01 – 2/2
5	SMITMSAFL	59689	9	47-55	5 publications, tested in 6 T cell assays and 2 MHC ligand assays	T cell assays:<br>IFNγ release – 0/6<br>MHC ligand assays:<br>HLA-A*02:01 – 2/2
6	SMVISLLSM	59769	9	40-48	3 publications, tested in 1 T cell assay and 3 MHC ligand assays	T cell assays:<br>IFNγ release – 0/1<br>MHC ligand assays:<br>HLA-A*02:01 – 0/2<br>HLA-B*15:03 – 1/1
7	TKTTSDYQDSDVSQ	64687	14	160-173	Tested in 1 T cell assay	IFNg release – 1/1
8	VLITWLIDYV	69551	10	139-148	4 publications, tested in 2 T cell assays and 2 MHC ligand assays	T cell assays:<br>IFNγ release – 0/2<br>MHC ligand assays:<br>HLA-A*02:01 – 2/2
9	AASSTHRKV	80156	9	79-87	2 publications, tested in 2 MHC ligand assays	HLA-A*02:01 – 2/2
10	AITDAAVAV	80297	9	69-77	2 publications, tested in 2 MHC ligand assays	HLA-A*02:01 – 2/2
11	AITDAAVAVA	80298	10	69-78	2 publications, tested in 2 MHC ligand assays	HLA-A*02:01 – 2/2
12	DKIQGKNKRKRVIGL	81087	15	21-35	2 publications, tested in 1 T	T cell assays:<br>IFNγ release – 0/1<br>MHC ligand assays:

					cell assay and 12 MHC ligand assays	HLA-DR13 – 1/1<br>HLA-DPB1*04:01 – 0/1<br>HLA-DPB1*04:02 – 0/1<br>HLA-DR1 – 0/1<br>HLA-DR11 – 0/1<br>HLA-DR15 – 0/1<br>HLA-DR3 – 0/1<br>HLA-DR4 – 0/1<br>HLA-DR7 – 0/1<br>HLA-DRB3 – 0/1<br>HLA-DRB4 – 0/1<br>HLA-DRB5 – 0/1
13	EAAITDAAV	81375	9	67-75	2 publications, tested in 2 MHC ligand assays	HLA-A*02:01 – 2/2
14	FLIVRLNQCM	82205	10	54-63	2 publications, tested in 2 MHC ligand assays	HLA-A*02:01 – 2/2
15	GLCIRISMV	82727	9	34-42	2 publications, tested in 2 MHC ligand assays	HLA-A*02:01 – 2/2
16	GLCIRISMVI	82728	10	34-43	2 publications, tested in 2 MHC ligand assays	HLA-A*02:01 – 2/2
17	ITWLIDYVEDTWGSD	84055	15	141-155	2 publications, tested in 1 T cell assay and 12 MHC ligand assays	T cell assays:<br>IFNγ release – 0/1<br>MHC ligand assays:<br>HLA-DR15 – 1/1<br>HLA-DR3 – 1/1<br>HLA-DR4 – 1/1<br>HLA-DPB1*04:01 – 0/1<br>HLA-DPB1*04:02 – 0/1<br>HLA-DR1 – 0/1<br>HLA-DR11 – 0/1<br>HLA-DR13 – 0/1<br>HLA-DR7 – 0/1<br>HLA-DRB3 – 0/1<br>HLA-DRB4 – 0/1<br>HLA-DRB5 – 0/1
18	LLSMITMSA	85522	9	45-53	2 publications, tested in 2	HLA-A*02:01 – 2/2

					MHC ligand assays	
19	LSMITMSAFLIVRLN	85862	15	46-60	2 publications, tested in 21 T cell assays and 12 MHC ligand assays	T cell assays:<br>IFNγ release – 20/21<br>MHC ligand assays:<br>HLA-DPB1*04:01 – 1/1<br>HLA-DPB1*04:02 – 1/1<br>HLA-DR1 – 1/1<br>HLA-DR11 – 1/1<br>HLA-DR13 – 1/1<br>HLA-DR15 – 1/1<br>HLA-DR3 – 1/1<br>HLA-DR7 – 1/1<br>HLA-DRB3 – 1/1<br>HLA-DRB4 – 1/1<br>HLA-DRB5 – 1/1<br>HLA-DR4 – 0/1
20	MITMSAFLI	86308	9	48-56	3 publications, tested in 3 MHC ligand assays	HLA-A*02:01 – 2/2<br>Mamu-B*017:04 – 1/1
21	MITMSAFLIV	86309	10	48-57	2 publications, tested in 2 MHC ligand assays	HLA-A*02:01 – 2/2
22	MVISLLSMI	86598	9	41-49	2 publications, tested in 2 MHC ligand assays	HLA-A*02:01 – 2/2
23	NGLYYQGSCYILHSD	86731	15	101-115	2 publications, tested in 5 T cell assays and 12 MHC ligand assays	T cell assays:<br>IFNγ release – 4/5<br>MHC ligand assays:<br>HLA-DR1 – 1/1<br>HLA-DR13 – 1/1<br>HLA-DR15 – 1/1<br>HLA-DR3 – 1/1<br>HLA-DPB1*04:01 – 0/1<br>HLA-DPB1*04:02 – 0/1<br>HLA-DR11 – 0/1<br>HLA-DR4 – 0/1<br>HLA-DR7 – 0/1<br>HLA-DRB3 – 0/1<br>HLA-DRB4 – 0/1<br>HLA-DRB5 – 0/1
24	QTSVFSATV	87736	9	10-18	2 publications, tested in 2	HLA-A*02:01 – 2/2

					MHC ligand assays	
25	RISMVISLL	87942	9	38-46	2 publications, tested in 2 MHC ligand assays	HLA-A*02:01 – 2/2
26	RVIGLCIRI	88293	9	31-39	2 publications, tested in 2 MHC ligand assays	HLA-A*02:01 – 2/2
27	RVIGLCIRISMVISL	88294	15	31-45	2 publications, tested in 7 T cell assays and 12 MHC ligand assays	T cell assays:<br>IFNγ release – 6/7<br>MHC ligand assays:<br>HLA-DPB1*04:01 – 1/1<br>HLA-DPB1*04:02 – 1/1<br>HLA-DR1 – 1/1<br>HLA-DR15 – 1/1<br>HLA-DR3 – 1/1<br>HLA-DR4 – 1/1<br>HLA-DR7 – 1/1<br>HLA-DRB4 – 1/1<br>HLA-DR11 – 0/1<br>HLA-DR13 – 0/1<br>HLA-DRB3 – 0/1<br>HLA-DRB5 – 0/1
28	SLLSMITMSA	88825	10	44-53	2 publications, tested in 2 MHC ligand assays	HLA-A*02:01 – 2/2
29	SMITMSAFLI	88893	10	47-56	2 publications, tested in 2 MHC ligand assays	HLA-A*02:01 – 2/2
30	SMVISLLSMI	88912	10	40-49	2 publications, tested in 2 MHC ligand assays	HLA-A*02:01 – 2/2
31	SQEVRKYFCV	88990	10	172-181	2 publications, tested in 2 MHC ligand assays	HLA-A*02:01 – 2/2
32	TMSAFLIVRL	89603	10	50-59	2 publications, tested in 2	HLA-A*02:01 – 2/2

					MHC ligand assays	
33	VIGLCIRISM	90020	10	32-41	2 publications, tested in 2 MHC ligand assays	HLA-A*02:01 – 2/2
34	VISLLSMIT	90054	9	42-50	2 publications, tested in 2 MHC ligand assays	HLA-A*02:01 – 1/2
35	WLIDYVEDT	90588	9	143-151	2 publications, tested in 2 MHC ligand assays	HLA-A*02:01 – 2/2
36	YQLFSDAKA	90919	9	116-124	2 publications, tested in 2 MHC ligand assays	HLA-A*02:01 – 2/2
37	AAITDAAVAVAAASS	103005	15	68-82	Tested in 12 MHC ligand assays	HLA-DR1 – 1/1<br>HLA-DPB1*04:01 – 0/1<br>HLA-DPB1*04:02 – 0/1<br>HLA-DR11 – 0/1<br>HLA-DR13 – 0/1<br>HLA-DR15 – 0/1<br>HLA-DR3 – 0/1<br>HLA-DR4 – 0/1<br>HLA-DR7 – 0/1<br>HLA-DRB3 – 0/1<br>HLA-DRB4 – 0/1<br>HLA-DRB5 – 0/1
38	AAVAVAAASSTHRKV	103009	15	73-87	Tested in 12 MHC ligand assays	HLA-DRB5 – 1/1<br>HLA-DPB1*04:01 – 0/1<br>HLA-DPB1*04:02 – 0/1<br>HLA-DR1 – 0/1<br>HLA-DR11 – 0/1<br>HLA-DR13 – 0/1<br>HLA-DR15 – 0/1<br>HLA-DR3 – 0/1<br>HLA-DR4 – 0/1<br>HLA-DR7 – 0/1<br>HLA-DRB3 – 0/1<br>HLA-DRB4 – 0/1
39	LIVRLNQCMSANEAA	103360	15	55-69	Tested in 12 MHC ligand assays	HLA-DR1 – 1/1<br>HLA-DPB1*04:01 – 0/1<br>HLA-DPB1*04:02 – 0/1<br>HLA-DR11 – 0/1

						HLA-DR13 – 0/1<br>HLA-DR15 – 0/1<br>HLA-DR3 – 0/1<br>HLA-DR4 – 0/1<br>HLA-DR7 – 0/1<br>HLA-DRB3 – 0/1<br>HLA-DRB4 – 0/1<br>HLA-DRB5 – 0/1
40	SAFLIVRLNQCMSAN	103581	15	52-66	Tested in 7 T cell assays and 12 MHC ligand assays	T cell assays:<br>IFNγ release – 7/7<br>MHC ligand assays:<br>HLA-DR1 – 1/1<br>HLA-DR11 – 1/1<br>HLA-DR13 – 1/1<br>HLA-DR15 – 1/1<br>HLA-DR3 – 1/1<br>HLA-DR4 – 1/1<br>HLA-DR7 – 1/1<br>HLA-DPB1*04:01 – 0/1<br>HLA-DPB1*04:02 – 0/1<br>HLA-DRB3 – 0/1<br>HLA-DRB4 – 0/1<br>HLA-DRB5 – 0/1
41	SCYILHSDYQLFSDA	103582	15	108-122	Tested in 12 MHC ligand assays	HLA-DR15 – 1/1<br>HLA-DR3 – 1/1<br>HLA-DRB3 – 1/1<br>HLA-DPB1*04:01 – 0/1<br>HLA-DPB1*04:02 – 0/1<br>HLA-DR1 – 0/1<br>HLA-DR11 – 0/1<br>HLA-DR13 – 0/1<br>HLA-DR4 – 0/1<br>HLA-DR7 – 0/1<br>HLA-DRB4 – 0/1<br>HLA-DRB5 – 0/1
42	SDYQLFSDAKANCTA	103588	15	114-128	Tested in 2 T cell assays and 12 MHC ligand assays	T cell assays:<br>IFNγ release – 2/2<br>MHC ligand assays:<br>HLA-DR3 – 1/1<br>HLA-DR4 – 1/1<br>HLA-DPB1*04:01 – 0/1<br>HLA-DPB1*04:02 – 0/1<br>HLA-DR1 – 0/1<br>HLA-DR11 – 0/1<br>HLA-DR13 – 0/1<br>HLA-DR15 – 0/1<br>HLA-DR7 – 0/1<br>HLA-DRB3 – 0/1<br>HLA-DRB4 – 0/1<br>HLA-DRB5 – 0/1

43	AFLIVRLNQCMSANEA	139996	16	53-68	Tested in 1 T cell assay	IFNγ release – 1/1
44	CYILHSDYQLFSDAKA	140011	16	109-124	Tested in 1 T cell assay	IFNγ release – 1/1
45	DKIQGKNKRKRVIGLC	140012	16	21-36	Tested in 1 T cell assay	IFNγ release – 1/1
46	EQTSVFSATVYGDKIQ	140022	16	9-24	Tested in 1 T cell assay	IFNγ release – 1/1
47	IRISMVISLLSMITMS	140037	16	37-52	Tested in 1 T cell assay	IFNγ release – 1/1
48	SSTTQYDHKESCNGLY	140072	16	89-104	Tested in 1 T cell assay	IFNγ release – 1/1
49	TVYGDKIQGKNKRKRV	140074	16	17-32	Tested in 1 T cell assay	IFNγ release – 1/1
50	EEQTSVFSA	189060	9	8-16	2 publications, tested in 2 MHC ligand assays	HLA-B*45:01 – 2/2
51	NEAAITDAAV	496800	10	66-75	Tested in 1 MHC ligand assay	HLA-B*45:01 – 1/1
52	ILHSDYQLF	83662	9	111-119	Tested in 1 MHC ligand assay	HLA-B*15:03 – 1/1
53	YILHSDYQL	90780	9	110-118	2 publications, tested in 2 MHC ligand assays	HLA-A*02:01 – 2/2
54	KANCTAESST	84178	10	123-132	2 publications, tested in 2 MHC ligand assays	HLA-A*02:01 – 2/2
55	DTWGSDGNPI	81309	10	150-159	2 publications, tested in 2 MHC ligand assays	HLA-A*02:01 – 2/2

Table S6. List of B cell epitopes (antigen A35R-A36R-A33R)

A35R-A36R-A33R (MPXV, VARV, and VACV) – Consensus sequence	Predicted linear epitopes	Position
>Consensus/1-185 Percentage Identity Consensus MMTPENDEEQTSVFSATVYGDKIQGKNKRKRVIGLCIRISMVISLLSMITMSAFLIVRLNQCMSANEAAITDAAVAVAAASSTHRKVASSTTQYDHKESCNGLYYQGSCYILHSDYQLFSDAKANCAAES	EEQTS	7-11
	STHRKVASSTTQYDHK	81-96

STLPNKSDVLTTWLIDYVEDTWGSDGNPITKTTSDYQDSDVSQ EVRKYFCVKTMN	PITKTTSDYQ DSDVSQEV	157-174

Table S7. List of T cell epitopes (antigen B6R-B7R-B5R**)** containing conserved amino acid sequences (origin VACV and VARV)

#	Epitope	Epitope ID	Length	Position	Source	Publications/Assays	Summary
1	CIDGKWNPILPTCVR	6410	15	225-239	Vaccinia	Tested in 2 T cell assays	Cytotoxicity – 2/2
2	FLISVIVLV	16674	9	292-300	Vaccinia	5 publications, tested in 2 T cell assays and 4 MHC ligand assays	T cell assays:<br>IFNγ release – 0/2<br>MHC ligand assays:<br>HLA-A*02:01 – 4/4
3	IIVALTIMGV	26716	10	281-290	Vaccinia	4 publications, tested in 2 T cell assays and 2 MHC ligand assays	T cell assays:<br>IFNγ release – 0/2<br>MHC ligand assays:<br>HLA-A*02:01 – 2/2
4	IMGVIFLISV	27497	10	287-296	Vaccinia	5 publications, tested in 2 T cell assays and 4 MHC ligand assays	T cell assays:<br>IFNγ release – 0/2<br>MHC ligand assays:<br>HLA-A*02:01 – 4/4
5	KMCTVSDYI	32294	9	74-82	Vaccinia	4 publications, tested in 4 T cell assays and 2 MHC ligand assays	T cell assays:<br>IFNγ release – 0/4<br>MHC ligand assays:<br>HLA-A*02:01 – 2/2
6	LTIMGVIFLI	39968	10	285-294	Vaccinia	4 publications, tested in 2 T cell assays and 2 MHC ligand assays	T cell assays:<br>IFNγ release – 0/2<br>MHC ligand assays:<br>HLA-A*02:01 – 2/2
7	NGLISGSTFSIGGVI	44027	15	193-207	Vaccinia	2 publications, tested in 1 T cell assay and 12 MHC ligand assays	T cell assays:<br>IFNγ release – 1/1<br>MHC ligand assays:<br>HLA-DR4 – 1/1<br>HLA-DR7 – 1/1<br>HLA-DPB1*04:01 – 0/1<br>HLA-DPB1*04:02 0/1<br>HLA-DR1 – 0/1<br>HLA-DR11 – 0/1<br>HLA-DR13 – 0/1<br>HLA-DR15 – 0/1<br>HLA-DR3 – 0/1<br>HLA-DRB3 – 0/1<br>HLA-DRB4 – 0/1<br>HLA-DRB5 – 0/1
8	SVVTLLCVLPAVVYS	62392	15	5-19	Vaccinia	2 publications, tested in 2 T cell assays	Cytotoxicity – 1/1<br>IFNγ release – 0/1
9	TIMGVIFLI	64382	9	286-294	Vaccinia	4 publications, tested in 2 T cell	T cell assays:<br>IFNγ release – 0/2

						assays and 2 MHC ligand assays	MHC ligand assays:<br>HLA-A*02:01 – 2/2
10	TKYFRCEEKNGNTSW	64699	15	105-119	Vaccinia	2 publications, tested in 1 T cell assay and 12 MHC ligand assays	T cell assays:<br>Cytotoxicity – 1/1<br>MHC ligand assays:<br>HLA-DR11 – 1/1<br>HLA-DPB1*04:01 – 0/1<br>HLA-DPB1*04:02 – 0/1<br>HLA-DR1 – 0/1<br>HLA-DR13 – 0/1<br>HLA-DR15 – 0/1<br>HLA-DR3 – 0/1<br>HLA-DR4 – 0/1<br>HLA-DR7 – 0/1<br>HLA-DRB3 – 0/1<br>HLA-DRB4 – 0/1<br>HLA-DRB5 – 0/1
11	TLLCVLPAV	64896	9	8-16	Vaccinia	4 publications, tested in 2 T cell assays and 2 MHC ligand assays	T cell assays:<br>IFNγ release – 0/2<br>MHC ligand assays:<br>HLA-A*02:01 – 2/2
12	TLLCVLPAVV	64897	10	8-17	Vaccinia	5 publications, tested in 2 T cell assays and 4 MHC ligand assays	T cell assays:<br>IFNγ release – 0/2<br>MHC ligand assays:<br>HLA-A*02:01 – 4/4
13	VLPAVVYST	69678	9	12-20	Vaccinia	4 publications, tested in 2 T cell assays and 2 MHC ligand assays	T cell assays:<br>IFNγ release – 0/2<br>MHC ligand assays:<br>HLA-A*02:01 – 2/2
14	ALTIMGVIFL	80386	10	284-293	Vaccinia	2 publications, tested in 2 MHC ligand assays	HLA-A*02:01 – 2/2
15	ASYISCTANSWNVIP	80503	15	166-180	Vaccinia	2 publications, tested in 8 T cell assays and 12 MHC ligand assays	T cell assays:<br>IFNγ release – 7/8<br>MHC ligand assays:<br>HLA-DPB1*04:01 – 1/1<br>HLA-DPB1*04:02 – 1/1<br>HLA-DR1 – 1/1<br>HLA-DR13 – 1/1<br>HLA-DR15 – 1/1<br>HLA-DR4 – 1/1<br>HLA-DR7 – 1/1<br>HLA-DRB3 – 1/1<br>HLA-DRB5 – 1/1<br>HLA-DR11 – 0/1<br>HLA-DR3 – 0/1<br>HLA-DRB4 – 0/1
16	ATYHIIIVAL	80541	10	276-285	Vaccinia	2 publications, tested in 2 MHC ligand assays	HLA-A*02:01 – 2/2

17	AVVYSTCTV	80587	9	15-23	Vaccinia	2 publications, tested in 2 MHC ligand assays	HLA-A*02:01 – 2/2
18	CIDGKWNPV	80683	9	225-233	Vaccinia	2 publications, tested in 2 MHC ligand assays	HLA-A*02:01 – 2/2
19	CIDGKWNPVL	80684	10	225-234	Vaccinia	2 publications, tested in 2 MHC ligand assays	HLA-A*02:01 – 2/2
20	CQQKCDMPSL	80785	10	182-191	Vaccinia	2 publications, tested in 2 MHC ligand assays	HLA-A*02:01 – 2/2
21	CTANSWNVI	80821	9	171-179	Vaccinia	2 publications, tested in 2 MHC ligand assays	HLA-A*02:01 – 2/2
22	CTVSDYISEL	80842	10	76-85	Vaccinia	2 publications, tested in 2 MHC ligand assays	HLA-A*02:01 – 2/2
23	CVLPAVVYST	80852	10	11-20	Vaccinia	2 publications, tested in 2 MHC ligand assays	HLA-A*02:01 – 2/2
24	DTVTCPNAECQPLQL	81308	15	121-135	Vaccinia	2 publications, tested in 1 T cell assay and 12 MHC ligand assays	T cell assays:<br>IFNγ release – 0/1<br>MHC ligand assays:<br>HLA-DRB4 – 1/1<br>HLA-DPB1*04:01 – 0/1<br>HLA-DPB1*04:02 – 0/1<br>HLA-DR1 – 0/1<br>HLA-DR11 – 0/1<br>HLA-DR13 – 0/1<br>HLA-DR15 – 0/1<br>HLA-DR3 – 0/1<br>HLA-DR4 – 0/1<br>HLA-DR7 – 0/1<br>HLA-DRB3 – 0/1<br>HLA-DRB5 – 0/1
25	EATYHIIIVA	81396	10	275-284	Vaccinia	2 publications, tested in 2 MHC ligand assays	HLA-A*02:01 – 2/2
26	ELYNKPLYEV	81691	10	84-93	Vaccinia	2 publications, tested in 2 MHC ligand assays	HLA-A*02:01 – 2/2
27	FTLTGSPSST	82427	10	215-224	Vaccinia	2 publications, tested in 2 MHC ligand assays	HLA-A*02:01 – 2/2
28	GLISGSTFSI	82745	10	194-203	Vaccinia	2 publications, tested in 2 MHC ligand assays	HLA-A*02:01 – 2/2

29	HIIIVALTI	83074	9	279-287	Vaccinia	2 publications, tested in 3 MHC ligand assays	HLA-A*02:01 – 0/2<br>HLA-A*32:01 – 1/1
30	HIIIVALTIM	83075	10	279-288	Vaccinia	2 publications, tested in 2 MHC ligand assays	HLA-A*02:01 – 2/2
31	HLSCKSGFTL	83138	10	208-217	Vaccinia	2 publications, tested in 2 MHC ligand assays	HLA-A*02:01 – 2/2
32	IVALTIMGV	84060	9	282-290	Vaccinia	2 publications, tested in 2 MHC ligand assays	HLA-A*02:01 – 2/2
33	KLTSTETSF	84538	9	30-38	Vaccinia	Tested in 1 MHC ligand assays	HLA-B*15:03 – 1/1
34	KTISVVTLL	84786	9	2-10	Vaccinia	2 publications, tested in 2 MHC ligand assays	HLA-A*02:01 – 2/2
35	KWKYENPCK	84906	9	65-73	Vaccinia	Tested in 5 MHC ligand assays	HLA-A*30:01 – 1/1<br>HLA-B*15:03 – 1/1<br>HLA-A*32:01 – 0/1<br>HLA-B*08:01 – 0/1<br>HLA-B*15:01 – 0/1
36	LISGSTFSI	85336	9	195-203	Vaccinia	2 publications, tested in 2 MHC ligand assays	HLA-A*02:01 – 2/2
37	LLCVLPAVV	85430	9	9-17	Vaccinia	2 publications, tested in 2 MHC ligand assays	HLA-A*02:01 – 2/2
38	LTIMGVIFL	85966	9	285-293	Vaccinia	2 publications, tested in 2 MHC ligand assays	HLA-A*02:01 – 2/2
39	MKTISVVTLLCVLPA	86334	15	1-15	Vaccinia	2 publications, tested in 10 T cell assays and 12 MHC ligand assays	T cell assays:<br>IFNγ release – 9/10<br>MHC ligand assays:<br>HLA-DPB1*04:01 – 1/1<br>HLA-DPB1*04:02 – 1/1<br>HLA-DR1 – 1/1<br>HLA-DR11 – 1/1<br>HLA-DR13 – 1/1<br>HLA-DR15 – 1/1<br>HLA-DR3 – 1/1<br>HLA-DR4 – 1/1<br>HLA-DR7 – 1/1<br>HLA-DRB3 – 1/1<br>HLA-DRB4 – 1/1<br>HLA-DRB5 – 0/1
40	MTLSCNGET	86565	9	97-105	Vaccinia	2 publications, tested in 2 MHC ligand assays	HLA-A*02:01 – 2/2

41	NTSWNDTVT	87107	9	116-124	Vaccinia	2 publications, tested in 2 MHC ligand assays	HLA-A*02:01 – 2/2
42	PAVVYSTCTV	87199	10	14-23	Vaccinia	2 publications, tested in 2 MHC ligand assays	HLA-A*02:01 – 2/2
43	PLYEVNSTM	87345	9	89-97	Vaccinia	2 publications, tested in 2 MHC ligand assays	HLA-A*02:01 – 2/2
44	PLYEVNSTMT	87346	10	89-98	Vaccinia	2 publications, tested in 2 MHC ligand assays	HLA-A*02:01 – 2/2
45	QLEHGSCQPV	87612	10	134-143	Vaccinia	2 publications, tested in 2 MHC ligand assays	HLA-A*02:01 – 2/2
46	QPLQLEHGSCQPVKE	87655	15	131-145	Vaccinia	2 publications, tested in 1 T cell assay and 12 MHC ligand assays	T cell assays:<br>IFNγ release – 0/1<br>MHC ligand assays:<br>HLA-DR1 – 1/1<br>HLA-DPB1*04:01 – 0/1<br>HLA-DPB1*04:02 – 0/1<br>HLA-DR11 – 0/1<br>HLA-DR13 – 0/1<br>HLA-DR15 – 0/1<br>HLA-DR3 – 0/1<br>HLA-DR4 – 0/1<br>HLA-DR7 – 0/1<br>HLA-DRB3 – 0/1<br>HLA-DRB4 – 0/1<br>HLA-DRB5 – 0/1
47	QPVKEKYSFGEYMTI	87661	15	141-155	Vaccinia	2 publications, tested in 1 T cell assay and 12 MHC ligand assays	T cell assays:<br>IFNγ release – 0/1<br>MHC ligand assays:<br>HLA-DPB1*04:01 – 1/1<br>HLA-DPB1*04:02 – 1/1<br>HLA-DR13 – 1/1<br>HLA-DR15 – 1/1<br>HLA-DR3 – 1/1<br>HLA-DR1 – 0/1<br>HLA-DR11 – 0/1<br>HLA-DR4 – 0/1<br>HLA-DR7 – 0/1<br>HLA-DRB3 – 0/1<br>HLA-DRB4 – 0/1<br>HLA-DRB5 – 0/1
48	RTNEEFDPV	88260	9	239-247	Vaccinia	2 publications, tested in 2 MHC ligand assays	HLA-A*02:01 – 2/2

49	SLEATYHII	88786	9	273-281	Vaccinia	2 publications, tested in 2 MHC ligand assays	HLA-A*02:01 – 2/2
50	SLEATYHIII	88787	10	273-282	Vaccinia	2 publications, tested in 2 MHC ligand assays	HLA-A*02:01 – 2/2
51	TINCDVGYEV	89490	10	154-163	Vaccinia	2 publications, tested in 2 MHC ligand assays	HLA-A*02:01 – 2/2
52	TISVVTLLCV	89501	10	3-12	Vaccinia	2 publications, tested in 2 MHC ligand assays	HLA-A*02:01 – 2/2
53	TVSDYISEL	89811	9	77-85	Vaccinia	2 publications, tested in 2 MHC ligand assays	HLA-A*02:01 – 2/2
54	VIFLISVIVL	90018	10	290-299	Vaccinia	2 publications, tested in 2 MHC ligand assays	HLA-A*02:01 – 2/2
55	VTLLCVLPA	90449	9	7-15	Vaccinia	2 publications, tested in 2 MHC ligand assays	HLA-A*02:01 – 2/2
56	VTLLCVLPAV	90450	10	7-16	Vaccinia	2 publications, tested in 2 MHC ligand assays	HLA-A*02:01 – 2/2
57	VVTLLCVLPA	90519	10	6-15	Vaccinia	2 publications, tested in 2 MHC ligand assays	HLA-A*02:01 – 2/2
58	VVYSTCTVPT	90523	10	16-25	Vaccinia	2 publications, tested in 2 MHC ligand assays	HLA-A*02:01 – 2/2
59	YISELYNKPL	90789	10	81-90	Vaccinia	2 publications, tested in 2 MHC ligand assays	HLA-A*02:01 – 2/2
60	CDVGYEVIGASYISC	103081	15	157-171	Vaccinia	Tested in 12 MHC ligand assays	HLA-DR1 – 1/1<br>HLA-DR3 – 1/1<br>HLA-DR4 – 1/1<br>HLA-DR7 – 1/1<br>HLA-DPB1*04:01 – 0/1<br>HLA-DPB1*04:02 – 0/1<br>HLA-DR11 – 0/1<br>HLA-DR13 – 0/1<br>HLA-DR15 – 0/1<br>HLA-DRB3 – 0/1<br>HLA-DRB4 – 0/1<br>HLA-DRB5 – 0/1
61	DKWKYENPCKKMCTV	103108	15	64-78	Vaccinia	Tested in 12 MHC ligand assays	HLA-DRB5 – 1/1<br>HLA-DPB1*04:01 – 0/1<br>HLA-DPB1*04:02 – 0/1<br>HLA-DR1 – 0/1<br>HLA-DR11 – 0/1

							HLA-DR13 – 0/1<br>HLA-DR15 – 0/1<br>HLA-DR3 – 0/1<br>HLA-DR4 – 0/1<br>HLA-DR7 – 0/1<br>HLA-DRB3 – 0/1<br>HLA-DRB4 – 0/1
62	EKYSFGEYMTINCDV	103142	15	145-159	Vaccinia	Tested in 12 MHC ligand assays	HLA-DR1 – 1/1<br>HLA-DR13 – 1/1<br>HLA-DPB1*04:01 – 0/1<br>HLA-DPB1*04:02 – 0/1<br>HLA-DR11 – 0/1<br>HLA-DR15 – 0/1<br>HLA-DR3 – 0/1<br>HLA-DR4 – 0/1<br>HLA-DR7 – 0/1<br>HLA-DRB3 – 0/1<br>HLA-DRB4 – 0/1<br>HLA-DRB5 – 0/1
63	EVIGASYISCTANSW	103155	15	162-176	Vaccinia	Tested in 12 MHC ligand assays	HLA-DR1 – 1/1<br>HLA-DR4 – 1/1<br>HLA-DPB1*04:01 – 0/1<br>HLA-DPB1*04:02 – 0/1<br>HLA-DR11 – 0/1<br>HLA-DR13 – 0/1<br>HLA-DR15 – 0/1<br>HLA-DR3 – 0/1<br>HLA-DR7 – 0/1<br>HLA-DRB3 – 0/1<br>HLA-DRB4 – 0/1<br>HLA-DRB5 – 0/1
64	GEYMTINCDVGYEVI	103204	15	150-164	Vaccinia	Tested in 12 MHC ligand assays	HLA-DR1 – 1/1<br>HLA-DR15 – 1/1<br>HLA-DRB3 – 1/1<br>HLA-DPB1*04:01 – 0/1<br>HLA-DPB1*04:02 – 0/1<br>HLA-DR11 – 0/1<br>HLA-DR13 – 0/1<br>HLA-DR3 – 0/1<br>HLA-DR4 – 0/1<br>HLA-DR7 – 0/1<br>HLA-DRB4 – 0/1<br>HLA-DRB5 – 0/1
65	GGVIHLSCKSGFILT	103218	15	204-218	Vaccinia	Tested in 9 T cell assays and 12 MHC ligand assays	T cell assays:<br>IFNγ release – 9/9<br>MHC ligand assays:<br>HLA-DPB1*04:02 – 1/1<br>HLA-DR1 – 1/1<br>HLA-DR11 – 1/1<br>HLA-DR13 – 1/1

							HLA-DR15 – 1/1<br>HLA-DR3 – 1/1<br>HLA-DR4 – 1/1<br>HLA-DR7 – 1/1<br>HLA-DRB4 – 1/1<br>HLA-DRB5 – 1/1<br>HLA-DPB1*04:01 – 0/1<br>HLA-DRB3 – 0/1
66	GKWNPVLPICVRTNE	103225	15	228-242	Vaccinia	Tested in 12 MHC ligand assays	HLA-DR15 – 1/1<br>HLA-DR7 – 1/1<br>HLA-DPB1*04:01 – 0/1<br>HLA-DPB1*04:02 – 0/1<br>HLA-DR1 – 0/1<br>HLA-DR11 – 0/1<br>HLA-DR13 – 0/1<br>HLA-DR3 – 0/1<br>HLA-DR4 – 0/1<br>HLA-DRB3 – 0/1<br>HLA-DRB4 – 0/1<br>HLA-DRB5 – 0/1
67	ISVVTLLCVLPAVVY	103288	15	4-18	Vaccinia	Tested in 10 T cell assays and 12 MHC ligand assays	T cell assays:<br>IFNγ release – 10/10<br>MHC ligand assays:<br>HLA-DR1 – 1/1<br>HLA-DR11 – 1/1<br>HLA-DR13 – 1/1<br>HLA-DR3 – 1/1<br>HLA-DR4 – 1/1<br>HLA-DPB1*04:01 – 0/1<br>HLA-DPB1*04:02 – 0/1<br>HLA-DR15 – 0/1<br>HLA-DR7 – 0/1<br>HLA-DRB3 – 0/1<br>HLA-DRB4 – 0/1<br>HLA-DRB5 – 0/1
68	IVLVCSCDKNNDCSC	103295	15		Vaccinia	Tested in 12 MHC ligand assays	HLA-DR4 – 1/1<br>HLA-DRB4 – 1/1<br>HLA-DPB1*04:01 – 0/1<br>HLA-DPB1*04:02 – 0/1<br>HLA-DR1 – 0/1<br>HLA-DR11 – 0/1<br>HLA-DR13 – 0/1<br>HLA-DR15 – 0/1<br>HLA-DR3 – 0/1<br>HLA-DR7 – 0/1<br>HLA-DRB3 – 0/1<br>HLA-DRB5 – 0/1
69	KDVVQYEQEIESLEA	103302	15	262-276	Vaccinia	Tested in 12 MHC ligand assays	HLA-DR11 – 1/1<br>HLA-DR15 – 1/1<br>HLA-DR3 – 1/1

							HLA-DPB1*04:01 – 0/1<br>HLA-DPB1*04:02 – 0/1<br>HLA-DR1 – 0/1<br>HLA-DR13 – 0/1<br>HLA-DR4 – 0/1<br>HLA-DR7 – 0/1<br>HLA-DRB3 – 0/1<br>HLA-DRB4 – 0/1<br>HLA-DRB5 – 0/1
70	KPLYEVNSTMTLSCN	103319	15	88-102	Vaccinia	Tested in 12 MHC ligand assays	HLA-DR11 – 1/1<br>HLA-DR4 – 1/1<br>HLA-DR7 – 1/1<br>HLA-DPB1*04:01 – 0/1<br>HLA-DPB1*04:02 – 0/1<br>HLA-DR1 – 0/1<br>HLA-DR13 – 0/1<br>HLA-DR15 – 0/1<br>HLA-DR3 – 0/1<br>HLA-DRB3 – 0/1<br>HLA-DRB4 – 0/1<br>HLA-DRB5 – 0/1
71	LCVLPAVVYSTCTVP	103341	15	10-24	Vaccinia	Tested in 12 MHC ligand assays	HLA-DR1 – 1/1<br>HLA-DR15 – 1/1<br>HLA-DR3 – 1/1<br>HLA-DR4 – 1/1<br>HLA-DR7 – 1/1<br>HLA-DPB1*04:01 – 0/1<br>HLA-DPB1*04:02 – 0/1<br>HLA-DR11 – 0/1<br>HLA-DR13 – 0/1<br>HLA-DRB3 – 0/1<br>HLA-DRB4 – 0/1<br>HLA-DRB5 – 0/1
72	NSWNVIPSCQQKCDI	103450	15	174-188	Vaccinia	Tested in 2 T cell assays and 12 MHC ligand assays	T cell assays:<br>IFNγ release – 2/2<br>MHC ligand assays:<br>HLA-DR11 – 1/1<br>HLA-DR4 – 1/1<br>HLA-DPB1*04:01 – 0/1<br>HLA-DPB1*04:02 – 0/1<br>HLA-DR1 – 0/1<br>HLA-DR13 – 0/1<br>HLA-DR15 – 0/1<br>HLA-DR3 – 0/1<br>HLA-DR7 – 0/1<br>HLA-DRB3 – 0/1<br>HLA-DRB4 – 0/1<br>HLA-DRB5 – 0/1
73	PAVVYSTCTVPTMNN	103461	15	14-28	Vaccinia	Tested in 12 MHC ligand assays	HLA-DR13 – 1/1<br>HLA-DR3 – 1/1

							HLA-DR4 – 1/1<br>HLA-DPB1*04:01 – 0/1<br>HLA-DPB1*04:02 – 0/1<br>HLA-DR1 – 0/1<br>HLA-DR11 – 0/1<br>HLA-DR15 – 0/1<br>HLA-DR7 – 0/1<br>HLA-DRB3 – 0/1<br>HLA-DRB4 – 0/1<br>HLA-DRB5 – 0/1
74	QEIESLEATYHIIIV	103517	15	269-283	Vaccinia	Tested in 6 T cell assays and 12 MHC ligand assays	T cell assays:<br>IFNγ release – 6/6<br>MHC ligand assays:<br>HLA-DR1 – 1/1<br>HLA-DR15 – 1/1<br>HLA-DR3 – 1/1<br>HLA-DR7 – 1/1<br>HLA-DPB1*04:01 – 0/1<br>HLA-DPB1*04:02 – 0/1<br>HLA-DR11 – 0/1<br>HLA-DR13 – 0/1<br>HLA-DR4 – 0/1<br>HLA-DRB3 – 0/1<br>HLA-DRB4 – 0/1<br>HLA-DRB5 – 0/1
75	SDYISELYNKPLYEV	103586	15	79-93	Vaccinia	Tested in 2 T cell assays and 12 MHC ligand assays	T cell assays:<br>IFNγ release – 2/2<br>MHC ligand assays:<br>HLA-DPB1*04:02 – 1/1<br>HLA-DR15 – 1/1<br>HLA-DPB1*04:01 – 0/1<br>HLA-DR1 – 0/1<br>HLA-DR11 – 0/1<br>HLA-DR13 – 0/1<br>HLA-DR3 – 0/1<br>HLA-DR4 – 0/1<br>HLA-DR7 – 0/1<br>HLA-DRB3 – 0/1<br>HLA-DRB4 – 0/1<br>HLA-DRB5 – 0/1
76	SGFILTGSPSSTCID	103593	15	213-227	Vaccinia	Tested in 12 MHC ligand assays	HLA-DR4 – 1/1<br>HLA-DR7 – 1/1<br>HLA-DPB1*04:01 – 0/1<br>HLA-DPB1*04:02 – 0/1<br>HLA-DR1 – 0/1<br>HLA-DR11 – 0/1<br>HLA-DR13 – 0/1<br>HLA-DR15 – 0/1<br>HLA-DR3 – 0/1<br>HLA-DRB3 – 0/1<br>HLA-DRB4 – 0/1

							HLA-DRB5 – 0/1
77	TCIDGKWNPVLPICV	103637	15	224-238	Vaccinia	Tested in 12 MHC ligand assays	HLA-DR4 – 1/1<br>HLA-DR7 – 1/1<br>HLA-DPB1*04:01 – 0/1<br>HLA-DPB1*04:02 – 0/1<br>HLA-DR1 – 0/1<br>HLA-DR11 – 0/1<br>HLA-DR13 – 0/1<br>HLA-DR15 – 0/1<br>HLA-DR3 – 0/1<br>HLA-DRB3 – 0/1<br>HLA-DRB4 – 0/1<br>HLA-DRB5 – 0/1
78	TDLSKLSKDVVQYEQ	103639	15	255-269	Vaccinia	Tested in 12 MHC ligand assays	HLA-DR3 – 1/1<br>HLA-DPB1*04:01 – 0/1<br>HLA-DPB1*04:02 – 0/1<br>HLA-DR1 – 0/1<br>HLA-DR11 – 0/1<br>HLA-DR13 – 0/1<br>HLA-DR15 – 0/1<br>HLA-DR4 – 0/1<br>HLA-DR7 – 0/1<br>HLA-DRB3 – 0/1<br>HLA-DRB4 – 0/1<br>HLA-DRB5 – 0/1
79	TSFNNNQKVTFTCDQ	103670	15	36-50	Vaccinia	Tested in 12 MHC ligand assays	HLA-DR13 – 1/1<br>HLA-DR3 – 1/1<br>HLA-DPB1*04:01 – 0/1<br>HLA-DPB1*04:02 – 0/1<br>HLA-DR1 – 0/1<br>HLA-DR11 – 0/1<br>HLA-DR15 – 0/1<br>HLA-DR4 – 0/1<br>HLA-DR7 – 0/1<br>HLA-DRB3 – 0/1<br>HLA-DRB4 – 0/1<br>HLA-DRB5 – 0/1
80	VTLLCVLPAVVYSTC	103719	15	7-21	Vaccinia	Tested in 12 MHC ligand assays	HLA-DPB1*04:02 – 1/1<br>HLA-DR1 – 1/1<br>HLA-DR13 – 1/1<br>HLA-DR15 – 1/1<br>HLA-DR3 – 1/1<br>HLA-DRB3 – 1/1<br>HLA-DRB4 – 1/1<br>HLA-DRB5 – 1/1<br>HLA-DPB1*04:01 – 0/1<br>HLA-DR11 – 0/1<br>HLA-DR4 – 0/1<br>HLA-DR7 – 0/1

81	AKLTSTETSFNNNQKV	139997	16	29-44	Vaccinia	Tested in 1 T cell assay	IFNγ release – 1/1
82	CETDKWKYENPCKKMC	140004	16	61-76	Vaccinia	Tested in 1 T cell assay	IFNγ release – 1/1
83	CTVPTMNNAKLTSTET	140009	16	21-36	Vaccinia	Tested in 1 T cell assay	IFNγ release – 1/1
84	FSIGGVIHLSCKSGFI	140025	16	201-216	Vaccinia	Tested in 1 T cell assay	IFNγ release – 1/1
85	GVIFLISVIVLVCSCD	140030	16	289-304	Vaccinia	Tested in 1 T cell assay	IFNγ release – 1/1
86	IIVALTIMGVIFLISV	140036	16	281-296	Vaccinia	Tested in 1 T cell assay	IFNγ release – 1/1
87	ITINCDVGYEVIGASY	140038	16	153-168	Vaccinia	Tested in 1 T cell assay	IFNγ release – 1/1
88	LYNKPLYEVNSTMTLS	140052	16	85-100	Vaccinia	Tested in 1 T cell assay	IFNγ release – 1/1
89	PNAVCETDKWKYENPC	140061	16	57-72	Vaccinia	Tested in 1 T cell assay	IFNγ release – 1/1
90	SGSTFSIGGVIHLSCK	140069	16	197-212	Vaccinia	Tested in 1 T cell assay	IFNγ release – 1/1
91	VYSTCTVPTMNNAKLT	140078	16	17-32	Vaccinia	Tested in 1 T cell assay	[illegible]
92	CSCDKNNDQY	189049	10	301-310	Vaccinia	Tested in 1 MHC ligand assay	HLA-A*01:01 – 1/1
93	VSDYISELYNKPLYE	874748	15	78-92	Vaccinia	Tested in 1 MHC ligand assay	HLA-DPB1*04:01 – 1/1
94	GVIFLISVIVLVCSCN	140031	16	289-304	Variola	Tested in 1 T cell assay	IFNγ release – 1/1

Table S8. List of B cell epitopes (antigen B6R-B7R-B5R)

B6R-B7R-B5R (MPXV, VARV, and VACV) – Consensus sequence	Predicted linear epitopes	Position
>Consensus/1-317 Percentage Identity Consensus MKTISVVTLLCVLPAVVYSTCTVPTMNNAKLTSTETSFNDKQK VTFTCDQGYHSLDPNAVCETDKWKYENPCKKMCTVSDYVSE LYNKPLYEVNSTMTLSCNGETKYFRCEEKNGNTSWNDTVTCP NAECQPLQLEHGSCQPVKEKYSFGEYMTINCDVGYEVIGASYI SCTANSWNVIPSCQQKCDIPSLSNGLISGSTFSIGGVIHLSCKSGF TLTGSPSSTCIDGKWNPILPTCVRSNEEFDPVDDGPDDETDLSK LSKDVVQYEQEIESLEATYHIIIVALTIMGVIFLISVIVLVCSCDK NNDQYKFHKLLP	VSELYNKPLY	81-90
	EEKNGNTSW NDTV	110-122
	PDDETDLSKL SKD	250-262

Table S9. List of T cell epitopes (antigen M1R-M1R-L1R**)** containing conserved amino acid sequences (origin VACV)

#	Epitope	Epitope ID	Length	Position	Publications/Assays	Summary
1	AALFMYYAK	275	9	197-205	Tested in 5 MHC ligand assays	HLA-A*03:01 – 1/1<br>HLA-A*11:01 – 1/1<br>HLA-A*31:01 – 1/1<br>HLA-A*68:01 – 1/1<br>HLA-A*33:01 – 0/1
2	AALFMYYAKR	276	10	197-206	Tested in 5 MHC ligand assays	HLA-A*03:01 – 1/1<br>HLA-A*11:01 – 1/1<br>HLA-A*31:01 – 1/1<br>HLA-A*33:01 – 1/1<br>HLA-A*68:01 – 1/1
3	ALFMYYAKR	2543	9	198-206	3 publications, tested in 5 T cell assays and 6 MHC ligand assays	T cell assays:<br>IFNγ release – 0/5<br>MHC ligand assays:<br>HLA-A*03:01 – 1/1<br>HLA-A*11:01 – 1/1<br>HLA-A*31:01 – 1/1<br>HLA-A*33:01 – 1/1<br>HLA-A*68:01 – 1/1<br>Patr-A*01:01 – 1/1
4	AMFTAALNI	3091	9	89-97	5 publications, tested in 2 T cell assays and 3 MHC ligand assays	T cell assays:<br>IFNγ release – 0/2<br>MHC ligand assays:<br>HLA-A*02:01 – 2/2

						HLA-A*32:01 – 1/1
5	APGSPTNLEF	3583	10	139-148	Tested in 5 MHC ligand assays	HLA-B*07:02 – 1/1<br>HLA-B*35:01 – 1/1<br>HLA-B*53:01 – 1/1<br>HLA-B*51:01 – 0/1<br>HLA-B*54:01 – 0/1
6	APKQVAGTGV	3623	10	175-184	Tested in 5 MHC ligand assays	HLA-B*07:02 – 1/1<br>HLA-B*54:01 – 1/1<br>HLA-B*35:01 – 0/1<br>HLA-B*51:01 – 0/1<br>HLA-B*53:01 – 0/1
7	AVLSAATETY	5452	10	67-76	3 publications, tested in 5 T cell assays and 5 MHC ligand assays	T cell assays:<br>IFNγ release – 0/5<br>MHC ligand assays:<br>HLA-A*03:01 – 1/1<br>HLA-A*11:01 – 1/1<br>HLA-A*68:01 – 1/1<br>HLA-A*31:01 – 0/1<br>HLA-A*33:01 – 0/1
8	DIEIGNFYIR	8617	10	35-44	Tested in 5 MHC ligand assays	HLA-A*03:01 – 1/1<br>HLA-A*31:01 – 1/1<br>HLA-A*33:01 – 1/1<br>HLA-A*68:01 – 1/1<br>HLA-A*11:01 – 0/1
9	EANASAQTK	11145	9	25-33	Tested in 5 MHC ligand assays	HLA-A*03:01 – 1/1

						HLA-A*11:01 – 1/1<br>HLA-A*68:01 – 1/1<br>HLA-A*31:01 – 0/1<br>HLA-A*33:01 – 0/1
10	EFINTGSSK	12016	9	147-155	Tested in 5 MHC ligand assays	HLA-A*33:01 – 1/1<br>HLA-A*68:01 – 1/1<br>HLA-A*03:01 – 0/1<br>HLA-A*11:01 – 0/1<br>HLA-A*31:01 – 0/1
11	FTSTNDKIK	18080	9	209-217	Tested in 5 MHC ligand assays	HLA-A*03:01 – 1/1<br>HLA-A*11:01 – 1/1<br>HLA-A*68:01 – 1/1<br>HLA-A*31:01 – 0/1<br>HLA-A*33:01 – 0/1
12	GSSKGNCAIK	22513	10	152-161	Tested in 5 MHC ligand assays	HLA-A*03:01 – 1/1<br>HLA-A*11:01 – 1/1<br>HLA-A*31:01 – 1/1<br>HLA-A*68:01 – 1/1<br>HLA-A*33:01 – 0/1
13	GTGVQFYMIVIGVIILAALFM	22681	21	281-201	Tested in 1 T cell assay and 1 MHC ligand assay	T cell assays:<br>IFNγ release – 0/1<br>MHC ligand assays:<br>HLA-DRB1*01:01 – 1/1
14	IILAALFMY	26560	9	194-202	Tested in 5 MHC ligand assays	HLA-A*03:01 – 1/1<br>HLA-A*11:01 – 1/1

						HLA-A*31:01 – 0/1<br>HLA-A*33:01 – 0/1<br>HLA-A*68:01 – 0/1
15	IILAALFMYY	26561	10	194-203	Tested in 5 MHC ligand assays	HLA-A*03:01 – 1/1<br>HLA-A*11:01 – 1/1<br>HLA-A*68:01 – 1/1<br>HLA-A*31:01 – 0/1<br>HLA-A*33:01 – 0/1
16	ILAALFMYY	26947	9	195-203	3 publications, tested in 7 T cell assays and 5 MHC ligand assays	T cell assays: IFNγ release – 0/7<br>MHC ligand assays:<br>HLA-A*03:01 – 1/1<br>HLA-A*11:01 – 1/1<br>HLA-A*68:01 – 1/1<br>HLA-A*31:01 – 0/1<br>HLA-A*33:01 – 0/1
17	IQTSVNTVVR	28239	10	97-106	3 publications, tested in 5 T cell assays and 5 MHC ligand assays	T cell assays: IFNγ release – 0/5<br>MHC ligand assays:<br>HLA-A*31:01 – 1/1<br>HLA-A*68:01 – 1/1<br>HLA-A*03:01 – 0/1<br>HLA-A*11:01 – 0/1<br>HLA-A*33:01 – 0/1
18	IVIGVIILA	29307	9	189-197	4 publications, tested in 2 T cell assays and 2 MHC ligand assays	T cell assays: IFNγ release – 0/2

						MHC ligand assays:<br>HLA-A*02:01 – 2/2
19	KALMQLTTK	29865	9	161-169	Tested in 5 MHC ligand assays	HLA-A*03:01 – 1/1<br>HLA-A*11:01 – 1/1<br>HLA-A*31:01 – 1/1<br>HLA-A*33:01 – 0/1<br>HLA-A*68:01 – 0/1
20	KATTQIAPK	29964	9	169-177	Tested in 5 MHC ligand assays	HLA-A*03:01 – 1/1<br>HLA-A*11:01 – 1/1<br>HLA-A*31:01 – 1/1<br>HLA-A*33:01 – 0/1<br>HLA-A*68:01 – 0/1
21	KIKLILANK	31291	9	215-223	2 publications, tested in 6 MHC ligand assays	HLA-A*03:01 – 1/1<br>HLA-A*11:01 – 1/1<br>HLA-A*30:01 – 1/1<br>HLA-A*31:01 – 1/1<br>HLA-A*33:01 – 0/1<br>HLA-A*68:01 – 0/1
22	KIQNVIIDECY	31403	11	127-137	2 publications, tested in 1 T cell assay and 1 MHC ligand assay	T cell assays:<br>IFNγ release – 1/1<br>MHC ligand assays:<br>HLA-DRB4*01:01 – 1/1
23	LAALFMYYA	34676	9	196-204	4 publications, tested in 2 T cell assays and 2 MHC ligand assays	T cell assays:<br>IFNγ release – 0/2<br>MHC ligand assays:

						HLA-A*02:01 – 2/2
24	LAALFMYYAK	34677	10	196-205	Tested in 6 MHC ligand assays	HLA-A*03:01 – 1/1<br>HLA-A*11:01 – 1/1<br>HLA-A*31:01 – 1/1<br>HLA-A*68:01 – 1/1<br>HLA-A*33:01 – 0/1<br>Patr-A*01:01 – 0/1
25	LFTSTNDKIK	36006	10	208-217	Tested in 5 MHC ligand assays	HLA-A*11:01 – 1/1<br>HLA-A*68:01 – 1/1<br>HLA-A*03:01 – 0/1<br>HLA-A*31:01 – 0/1<br>HLA-A*33:01 – 0/1
26	MLFTSTNDK	41940	9	207-215	3 publications, tested in 5 T cell assays and 5 MHC ligand assays	T cell assays:<br>IFNγ release – 0/5<br>MHC ligand assays:<br>HLA-A*03:01 – 1/1<br>HLA-A*11:01 – 1/1<br>HLA-A*68:01 – 1/1<br>HLA-A*31:01 – 0/1<br>HLA-A*33:01 – 0/1
27	NSSAVVDNK	45970	9	117-125	Tested in 5 MHC ligand assays	HLA-A*11:01 – 1/1<br>HLA-A*68:01 – 1/1<br>HLA-A*03:01 – 0/1<br>HLA-A*31:01 – 0/1<br>HLA-A*33:01 – 0/1

28	NTLSERISSK	46167	10	12-21	Tested in 5 MHC ligand assays	HLA-A*03:01 – 1/1<br>HLA-A*11:01 – 1/1<br>HLA-A*31:01 – 1/1<br>HLA-A*33:01 – 1/1<br>HLA-A*68:01 – 1/1
29	NTVVRDFENY	46311	10	102-111	Tested in 5 MHC ligand assays	HLA-A*11:01 – 1/1<br>HLA-A*68:01 – 1/1<br>HLA-A*03:01 – 0/1<br>HLA-A*31:01 – 0/1<br>HLA-A*33:01 – 0/1
30	QTSVNTVVR	52633	9	98-106	Tested in 5 MHC ligand assays	HLA-A*11:01 – 1/1<br>HLA-A*31:01 – 1/1<br>HLA-A*68:01 – 1/1<br>HLA-A*03:01 – 0/1<br>HLA-A*33:01 – 0/1
31	QTTVNTLSER	52649	10	8-17	Tested in 5 MHC ligand assays	HLA-A*03:01 – 1/1<br>HLA-A*11:01 – 1/1<br>HLA-A*31:01 – 1/1<br>HLA-A*33:01 – 1/1<br>HLA-A*68:01 – 1/1
32	QVAGTGVQFY	52676	10	178-187	Tested in 5 MHC ligand assays	HLA-A*03:01 – 1/1<br>HLA-A*11:01 – 1/1<br>HLA-A*68:01 – 1/1<br>HLA-A*31:01 – 0/1<br>HLA-A*33:01 – 0/1

33	RMLFTSTNDK	54917	10	206-215	Tested in 5 MHC ligand assays	HLA-A*03:01 – 1/1<br>HLA-A*11:01 – 1/1<br>HLA-A*31:01 – 1/1<br>HLA-A*68:01 – 1/1<br>HLA-A*33:01 – 0/1
34	SAVVDNKLK	57041	9	119-127	Tested in 5 MHC ligand assays	HLA-A*11:01 – 1/1<br>HLA-A*68:01 – 1/1<br>HLA-A*03:01 – 0/1<br>HLA-A*31:01 – 0/1<br>HLA-A*33:01 – 0/1
35	SPMVIATTDM	60160	10	239-248	Tested in 5 MHC ligand assays	HLA-B*07:02 – 1/1<br>HLA-B*35:01 – 1/1<br>HLA-B*53:01 – 1/1<br>HLA-B*54:01 – 1/1<br>HLA-B*51:01 – 0/1
36	SSAVVDNKLK	60916	10	118-127	Tested in 5 MHC ligand assays	HLA-A*03:01 – 1/1<br>HLA-A*11:01 – 1/1<br>HLA-A*31:01 – 1/1<br>HLA-A*68:01 – 1/1<br>HLA-A*33:01 – 0/1
37	SSKGNCAIK	61137	9	153-161	Tested in 5 MHC ligand assays	HLA-A*11:01 – 1/1<br>HLA-A*31:01 – 1/1<br>HLA-A*68:01 – 1/1<br>HLA-A*03:01 – 0/1<br>HLA-A*33:01 – 0/1

38	TLSERISSK	65058	9	13-21	Tested in 5 MHC ligand assays	HLA-A*03:01 – 1/1<br>HLA-A*11:01 – 1/1<br>HLA-A*31:01 – 1/1<br>HLA-A*68:01 – 1/1<br>HLA-A*33:01 – 0/1
39	TPEQKAYVPA	65559	10	80-89	Tested in 5 MHC ligand assays	HLA-B*07:02 – 1/1<br>HLA-B*54:01 – 1/1<br>HLA-B*35:01 – 0/1<br>HLA-B*51:01 – 0/1<br>HLA-B*53:01 – 0/1
40	TTVNTLSER	66897	9	9-17	Tested in 5 MHC ligand assays	HLA-A*11:01 – 1/1<br>HLA-A*31:01 – 1/1<br>HLA-A*33:01 – 1/1<br>HLA-A*68:01 – 1/1<br>HLA-A*03:01 – 0/1
41	TTYMDTFFR	66923	9	229-237	3 publications, tested in 5 T cell assays and 6 MHC ligand assays	T cell assays:<br>IFNγ release – 0/5<br>MHC ligand assays:<br>HLA-A*03:01 – 1/1<br>HLA-A*11:01 – 1/1<br>HLA-A*31:01 – 1/1<br>HLA-A*33:01 – 1/1<br>HLA-A*68:01 – 1/1<br>Patr-A*01:01 – 1/1
42	TVVRDFENY	67201	9	103-111	Tested in 5 MHC ligand assays	HLA-A*11:01 – 1/1

						HLA-A*68:01 – 1/1<br>HLA-A*03:01 – 0/1<br>HLA-A*31:01 – 0/1<br>HLA-A*33:01 – 0/1
43	VIILAALFMY	68993	10	193-202	Tested in 5 MHC ligand assays	HLA-A*03:01 – 1/1<br>HLA-A*11:01 – 1/1<br>HLA-A*31:01 – 0/1<br>HLA-A*33:01 – 0/1<br>HLA-A*68:01 – 0/1
44	VLSAATETY	69748	9	68-76	2 publications, tested in 2 T cell assays and 5 MHC ligand assays	T cell assays:<br>IFNγ release – 0/7<br>MHC ligand assays:<br>HLA-A*03:01 – 1/1<br>HLA-A*68:01 – 1/1<br>HLA-A*11:01 – 0/1<br>HLA-A*31:01 – 0/1<br>HLA-A*33:01 – 0/1
45	VPAMFTAAL	70248	9	87-95	9 publications, tested in 7 T cell assays and 24 MHC ligand assays	T cell assays:<br>IFNγ release – 0/2<br>MHC ligand assays:<br>HLA-B*07:02 – 2/2<br>HLA-A*02:06 – 1/1<br>HLA-A*69:01 – 1/1<br>HLA-B*08:01 – 1/1<br>HLA-B*15:17 – 1/1<br>HLA-B*35:01 – 1/1

						HLA-B*39:01 – 1/1<br>HLA-B*51:01 – 1/1<br>HLA-B7 – 1/1<br>HLA-A*01:01 – 0/1<br>HLA-A*02:01 – 0/1<br>HLA-A*03:01 – 0/1<br>HLA-A*11:01 – 0/1<br>HLA-A*30:01 – 0/1<br>HLA-A*31:01 – 0/1<br>HLA-B*15:01 – 0/1<br>HLA-B*27:05 – 0/1<br>HLA-B*40:01 – 0/1<br>HLA-B*46:01 – 0/1<br>HLA-B*48:01 – 0/1<br>HLA-B*57:01 – 0/1<br>HLA-B*58:01 – 0/1<br>HLA-C*05:01 – 0/1
46	VVRDFENYVK	71808	10	104-113	3 publications, tested in 5 T cell assays and 5 MHC ligand assays	T cell assays:<br>IFNγ release – 0/5<br>MHC ligand assays:<br>HLA-A*03:01 – 1/1<br>HLA-A*11:01 – 1/1<br>HLA-A*31:01 – 1/1<br>HLA-A*68:01 – 1/1<br>HLA-A*33:01 – 0/1
47	WTTYMDTFFR	73163	10	228-237	3 publications, tested in 5 T cell assays and 18 MHC ligand assays	T cell assays:<br>IFNγ release – 0/5

						MHC ligand assays:<br>HLA-A*11:01 – 1/1<br>HLA-A*31:01 – 1/1<br>HLA-A*33:01 – 1/1<br>HLA-A*68:01 – 1/1<br>H2-Db – 0/1<br>H2-Dd – 0/1<br>H2-Kb – 0/1<br>HLA-A*02:01 – 0/1<br>HLA-A*02:02 – 0/1<br>HLA-A*02:03 – 0/1<br>HLA-A*02:06 – 0/1<br>HLA-A*03:01 – 0/1<br>HLA-A*68:02 – 0/1<br>HLA-B*07:02 – 0/1<br>HLA-B*35:01 – 0/1<br>HLA-B*51:01 – 0/1<br>HLA-B*53:01 – 0/1<br>HLA-B*54:01 – 0/1
48	YMIVIGVIILAALFMYYAKRM	75069	21	187-207	Tested in 1 T cell assay and 1 MHC ligand assay	T cell assays:<br>IFNγ release – 0/1<br>MHC ligand assays:<br>HLA-DRB1*01:01 – 1/1
49	YSGLTPEQK	75745	9	76-84	Tested in 5 MHC ligand assays	HLA-A*11:01 – 1/1<br>HLA-A*68:01 – 1/1<br>HLA-A*03:01 – 0/1<br>HLA-A*31:01 – 0/1

						HLA-A*33:01 – 0/1
50	EQKAYVPAM	81769	9	82-90	Tested in 1 MHC ligand assays	HLA-B*15:01 – 1/1
51	TTKATTQIA	89748	9	167-175	Tested in 1 MHC ligand assays	HLA-A*30:01 – 1/1
52	AALFMYYAKRMLFTST	139994	16	197-212	Tested in 1 T cell assay	IFNγ release – 1/1
53	ANKENVHWTTYMDTFF	139998	16	221-236	Tested in 1 T cell assay	IFNγ release – 1/1
54	CNLTVKNMCSADADAQ	140007	16	49-64	Tested in 1 T cell assay	IFNγ release – 1/1
55	DTFFRTSPMVIATTDM	140016	16	233-248	Tested in 1 T cell assay	IFNγ release – 1/1
56	KATTQIAPRQVAGTGV	140042	16	169-184	Tested in 1 T cell assay	IFNγ release – 1/1
57	KCDIEIGNFYIRQNHG	140043	16	33-48	Tested in 1 T cell assay	IFNγ release – 1/1
58	KLEQEANASAQTKCDI	140047	16	21-36	Tested in 1 T cell assay	IFNγ release – 1/1
59	KRMLFTSTNDKIKLIL	140049	16	205-220	Tested in 1 T cell assay	IFNγ release – 1/1
60	MYYAKRMLFTSTNDKI	140055	16	201-216	Tested in 1 T cell assay	IFNγ release – 1/1
61	NCAIKALMQLTTKATT	140056	16	157-172	Tested in 1 T cell assay	IFNγ release – 1/1
62	NVHWTTYMDTFFRTSP	140058	16	225-240	Tested in 1 T cell assay	IFNγ release – 1/1
63	QIAPRQVAGTGVQFYM	140063	16	173-188	Tested in 1 T cell assay	IFNγ release – 1/1
64	QNVIIDECYGAPGSPT	140065	16	129-144	Tested in 1 T cell assay	IFNγ release – 1/1
65	TTYMDTFFRTSPMVIA	140073	16	229-244	Tested in 1 T cell assay	IFNγ release – 1/1
66	VIILAALFMYYAKRML	140076	16	193-208	Tested in 1 T cell assay	IFNγ release – 1/1
67	IGVIILAALFMYYAK	190248	15	191-205	Tested in 2 MHC ligand assays	HLA-DRB1*01:01 – 2/2

Table S10. List of B cell epitopes (antigen M1R-M1R-L1R)

M1R-M1R-L1R (MPXV, VARV, and VACV) – Consensus sequence	Predicted linear epitopes	Position
>Consensus/1-250 Percentage Identity Consensus MGAAASIQTTVNTLSERISSKLEQEANASAQTKCDIEIGNFYIRQ NHGCNITVKNMCSADADAQLDAVLSAATETYSGLTPEQKAYV PAMFTAALNIQTSVNTVVRDFENYVKQTCNSSAVVDNKLKIQ NVIIDECYGAPGSPTNLEFINTGSSKGNCAIKALMQLTTKATTQI	EANAS	24-28
	QTSVNT	97-102

APRQVAGTGVQFYMIVIGVIILAALFMYYAKRMLFTSTNDKIK LILANKENVHWTTYMDTFFRTSPMIIATTDIQN	APRQV	174-178

Table S11. Molecular docking results of the final vaccine constructs with MHC-I

Peptide	Cluster Size	Center Weighted Score	Lowest Energy
TLFPGDDDL	409	-759.2	-860.1
NTLSERISSK	198	-575.0	-596.6

Table S12. Molecular docking results of the final vaccine constructs with MHC-II

Peptide	Cluster Size	Center Weighted Score	Lowest Energy
FFIVVATAAVCLLFI	162	-990.6	-1074.9
LSMITMSAFLIVRLN	227	-1013.4	-1051.4
ASYISCTANSWNVIP	149	-932.3	-1096.5
KIQNVIIDECY	174	-960.8	-960.8
